# Supplementary material for: Pyrolysis-GCMS of Spirulina platensis: Evaluation of biomasses cultivated under autotrophic and mixotrophic conditions
Source: PLoS One. 2022 Oct 20;17(10):e0276317. doi: 10.1371/journal.pone.0276317 (PMC9584514; doi:10.1371/journal.pone.0276317)
Supplement: S1 File — 10.6084/m9.figshare.20278794. (DOCX) [file pone.0276317.s001.docx]

**Supporting Information**

**Pyrolysis-GCMS of *Spirulina platensis*:** [**evaluation of biomasses cultivated under**](https://www.sciencedirect.com/science/article/pii/S0016236116301132) **autotrophic and mixotrophic conditions**

Sueilha F. A. Paula^1¶^, Bruna M. E. Chagas^2¶*^, Maria I. B. Pereira^3&^, Adriano H. N. Rangel^3&^, Cristiane Sassi^4&^, Luiz H. F. Borba^3&^, Everaldo S. Santos^5&^, Estefani A. Asevedo^5&^, Fabiana R. A. Câmara ^3&^, Renata M. Araújo ^1¶^

http://[10.6084/m9.figshare.20278794](https://doi.org/10.6084/m9.figshare.20278794)

**S1 Table List of semi-quantified volatile compounds (% area) from pyrolysis of A, M2.5, M5, M10 at 450ºC.**

|  | **A** | **M2.5** | **M5** | **M10** | **Compounds** |
| --- | --- | --- | --- | --- | --- |
| **RT** | **Concentration (% area)** | | | | **Name** |
| 5.891 |  | 3,17 ± 0,35 |  |  | Acetonitrila (C₂H₃N) |
| 5.902 |  |  | 2,96 ± 0,00 |  | Isobutylene epoxide (C_4_H_8_) |
| 5.930 | 1,41 ± 0,01 |  |  | 1,63 ± 0,77 | 2-methyl-Propanal(C_4_H_8_O) |
| 8.103 | 1,63 ± 0,05 |  | 1,69 ± 0,06 | 2,08 ± 0,07 | 3-methyl-Butanal(C_5_H_10_O) |
| 8.282 | 1,66 ± 0,01 |  |  | 1,68 ± 0,09 | 2-methyl-Butanal(C_5_H1_0_O) |
| 9.454 | 5,33 ± 0,30 |  |  | 7,61 ± 0,13 | Etanoic acid(CH_3_COOH) |
| 10.827 | 1,88 ± 0,31 |  | 4,88 ± 0,28 | 10,80 ± 0,60 | 1-hydroxy-2-Propanone(C_3_H_6_O) |
| 11.076 |  |  |  |  | Toluene(C_7_H_8_) |
| 13.004 |  | 1,69 ± 0,25 | 1,00 ± 0,05 |  | 3-methyl-Butanenitrile(C_5_H_9_N) |
| 15.883 |  | 1,51 ± 0,4 | 2,51 ± 0,10 | 2,96 ± 0,13 | Pyrrole (C_4_H_5_N) |
| 18.817 |  |  | 1,84 ± 0,06 |  | 2-Cyclopenten-1-one(C_5_H_6_O) |
| 19.176 | 1,02 ± 0,01 | 2,72 ± 0,33 | 1,87 ± 0,06 | 0,92 ± 0,17 | 4-methyl-Pentanenitrile(C_6_H_11_N) |
| 20.564 |  |  |  | 0,70 ± 0,07 | 2-methyl-1H-Pyrrole(C_5_H_7_N) |
| 21.288 | 2,71 ± 0,92 |  |  | 4,44 ± 0,20 | 2-Furanmethanol(C_5_H_6_O) |
| 21.398 |  | 3,04 ± 0,10 |  |  | Acetamide(C_2_H_5_NO) |
| 21.763 |  |  |  | 1,45 ± 0,27 | 1-(acetyloxy)-2-Propanone(C_5_H_8_O) |
| 21.844 |  |  |  | 0,98 ± 0,42 | Crotonic acid(C_4_H_6_O_2_) |
| 24.910 |  |  |  | 1,69 ± 0,35 | 2-hydroxy-  2-Cyclopenten-1-one(C_5_H_6_O) |
| 27.618 |  |  |  | 0,93 ± 0,08 |  |
| 30.279 |  |  | 1,32 ± 0,05 | 2,16 ± 0,12 | 2-hydroxy-3-methyl-2-Cyclopenten-1-one (C_6_H_8_O_2_) |
| 32.151 | 2,60 ± 0,23 | 5,00 ± 0,37 | 3,88 ± 0,66 | 1,46 ± 0,31 | Phenol(C_6_H_6_O) |
| 32.835 |  |  | 0,87 ± 0,07 |  | 3-methyl-Butanamide(C_5_H_11_NO) |
| 34.834 |  |  |  | 1,31 ± 0,06 | 2-hydroxy-3-ethyl-2-Cyclopenten-  1-one(C_7_H_10_O) |
| 36.415 | 0,76 ± 0,18 | 1,06 ± 0,06 |  |  | 4-methyl-Phenol(C_21_H_28_O) |
| 37.971 |  | 0,96 ± 0,11 |  |  | Benzyl nitrile(C_8_H_7_N) |
| 38.354 |  |  |  | 3,84 ± 1,06 | Cyclopropyl carbinol(C_4_H_8_O) |
| 39.029 | 1,13 ± 0,18 | 1,63 ± 0,11 | 1,31 ± 0,13 | 0,83 ± 0,11 | Hexadecanamide(C_16_H_33_NO) |
| 40.463 | 0,94 ± 0,09 |  |  |  | Tetradecane(C_14_H_30_) |
| 40.490 |  | 0,95 ± 0,38 |  |  | Pentadecane(C_15_H_32_) |
| 40.652 |  |  |  |  | 9-Oxabicyclo[4.2.1]nonane(C_8_H_14_O) |
| 42.148 | 0,88 ± 0,47 |  |  |  | Dihydro-2H-Pyran-2,6(3H)-dione  (C_5_H_4_O_3_) |
| 42.247 | 1,54 ± 0,11 |  |  | 1,10 ± 0,01 | 2,5-Pyrrolidinedione(C_11_H_12_N_2_O_2_) |
| 43.561 | 1,38 ± 0,57 | 2,18 ± 0,01 | 1,99 ± 0,05 |  | Benzenepropanenitrile(C_9_H_9_N) |
| 43.751 |  |  | 1,02 ± 0,08 | 0,83 ± 0,05 | 1,4:3,6-Dianhydro-.alpha.-d-glucopyranose (C_6_H_8_O_4_) |
| 47.327 |  |  |  | 0,83 ± 0,16 | (S)-(+)-2',3'-Dideoxyribonolactone (C_5_H_8_O_3_) |
| 47.579 | 3,05 ± 0,13 | 5,28 ± 0,01 | 4,43 ± 0,37 | 2,07 ± 0,37 | Indole (C_8_H_7_N) |
| 49.015 | 1,23 ± 0,18 |  |  |  | 1,3-Cyclopentanedione(C_5_H_6_O_2_) |
| 49.058 |  | 1,59 ± 0,01 | 1,56 ± 0,07 | 0,81 ± 0,07 | 2,4-Dimethyl-2-oxazoline-4-methanol  (C_6_H_11_NO_2_) |
| 51.480 | 1,25 ± 0,11 | 1,45 ± 0,03 | 1,41 ± 0,08 | 0,74 ± 0,06 | 1,4-Cyclohexanedione (C_6_H_8_O_2_) |
| 53.123 | 9,96 ± 1,30 |  |  |  | Heneicosane (C_21_H_44_) |
| 53.142 |  | 4,47 ± 0,13 | 4,94 ± 0,13 |  | Heptadecane (C_17_H36) |
| 53.296 | 2,20 ± 0,30 |  |  |  | 1-[(1-Methyl-2-oxido-2-oxohydrazin)methyl]piperidine  (C_7_H_15_N_3_O_2_) |
| 53.334 |  | 3,00 ± 0,06 | 2,80 ± 0,03 | 1,20 ± 0,10 | 2,4-Dimethyl-4-hydroxymethyl-2-oxazoline (C_6_H_11_NO) |
| 53.474 |  |  | 0,92 ± 0,04 |  | 4-Amino-6-methyl-piperidin-2-one  (C_6_H_12_N_2_) |
| 55.607 | 2,27 ± 0,09 | 3,27 ± 0,16 | 2,86 ± 0,21 | 1,41 ± 0,12 | 1-(cyanoacetyl)-Piperidine  (C_8_H_12_N_2_O) |
| 56.389 |  | 1,35 ± 0,07 |  |  | N-(2-Cyanoacetyl)piperidine  (C_8_H_12_N_2_O) |
| 56.339 |  |  | 1,17 ± 0,08 |  | Pyrrolidin-2-one, 5-[2-propionylethyl]-(C_9_H_15_NO_2_) |
| 58.409 | 1,18 ± 0,02 |  | 1,32 ± 0,16 |  | 3,7,11,15-tetramethyl-2-Hexadecene (C_20_H_40_) |
| 58.780 | 1,91 ± 0,18 |  | 1,32 ± 0,01 | 1,05 ± 0,06 | 3,7,11,15-tetramethyl-2-Hexadecene isomer (C_20_H_40_) |
| 59.187 | 11,5 ± 1,59 | 6,73 ± 0,30 | 8,37 ± 0,69 | 7,52 ± 0,62 | 3,7,11,15-Tetramethyl-2-hexadecen-1-ol (C_20_H_40_O) |
| 59.355 |  |  | 0,69 ± 0,10 |  | 1-(5-methyl-2-propan-2-ylcyclohexyl)oxy-3-morpholin-4-yl-propan-2-ol  (C_17_H_33_NO_3_) |
| 59.477 |  | 0,86 ± 0,03 |  |  | N-(1,2-dihydro-4,6-dimethyl-2-oxo-3-pyridyl)-Acetamide (C_9_H_12_N2O_2_) |
| 59.993 | 1,34 ± 0,03 |  |  |  | Cyclohexadecane(C_16_H_32_) |
| 60.045 | 1,63 ± 0,06 |  |  |  | Palmitoyl chloride - (C_l1_6H_31_ClO) |
| 60.124 |  |  | 1,23 ± 0,12 |  | N,N-dimethyl-3-Octen-2-amine  (C_10_H_12_N) |
| 60.546 |  |  | 0,89 ± 0,10 |  | 2-(1-methylethyl)-Cyclohexanone (C_9_H_16_O) |
| 60.660 | 2,37 ± 0,28 | 1,46 ± 0,13 | 1,57 ± 0,30 | 1,32 ± 0,12 | m-Menth-1(7)-ene(C_10_H_18_) |
| 60.869 | 4,06 ± 0,58 | 2,51 ± 0,07 | 2,86 ± 0,38 | 2,42 ± 0,26 | Phytol (C_20_H_40_O) |
| 63.174 |  | 1,29 ± 0,04 | 1,10 ± 0,23 |  | 3-Ethyl-5-methylindolizidine (C_11_H_21_N) |
| 64.016 |  |  |  | 0,96 ± 0,42 | Cyclopent-3-ene-3-amino  (C_5_H_9_N) |
| 64.083 | 1,51 ± 0,12 |  |  |  | 3-methoxy-2-methyl-Phenol (C_8_H_10_O_2_) |
| 64.094 |  |  | 2,62 ± 0,08 |  | Methyl ester-Dodecanoic acid (C_13_H_26_O_2_) |
| 66.242 | 1,51 ± 0,24 | 1,89 ± 0,26 |  | 1,15 ± 0,14 | 1,2-Benzenedicarboxylic acid (C_16_H_22_O) |
| 66.606 | 1,39 ± 0,13 |  | 1,75 ± 0,23 |  | Hexadecanitrile (C_16_H_31_N) |
| 66.692 |  |  | 0,76 ± 0,04 |  | 4-Methyl Hydrazono Methyl-2,2,5,5-tetramethyl-3-imidazoline-1-oxyl (C_9_H_17_N_4_O) |
| 68.690 | 9,71 ± 1,86 |  |  | 9,15 ± 0,37 | Pentadecanoic acid (C_15_H_30_O_2_) |
| 68.901 | 11,02 ±1,86 |  |  |  | l-(+)-Ascorbic acid 2,6-dihexadecanoate (C_38_H_68_O_8_) |
| 71.299 |  | 1,59 ± 0,65 | 1,66 ± 0,45 |  | E-Phytol - (C_20_H_40_O) |
| 73.059 |  |  |  |  | 9-Octadecyne (C_18_H_34_) |
| 74.478 | 0,85 ± 0,13 |  |  |  | Oleic Acid (C_18_H_34_O_2_) |
| 74.702 | 2,02 ± 0,58 |  |  |  | 9,12-Octadecadienoic acid  (C_18_H_32_O_2_) |
| 77.925 | 8,04 ± 1,2 | 15,96 ± 1,14 | 15,68 ± 1,80 | 4,90 ± 0,81 | Hexadecanamide (C_16_H_33_NO) |

*Retention Time (RT),, ± standard error.

**S2 Table List of semi-quantified volatile compounds (% area) from pyrolysis of A, M2.5, M5, M10 at 550ºC.**

|  | **A** | **M2.5** | **M5** | **M10** | **Compounds** |
| --- | --- | --- | --- | --- | --- |
| **RT** | **Concentration (% area)** | | | | **Name** |
| 5,87 |  |  | 6,34 ± 0,70 |  | Acetonitrila (C₂H₃N) |
| 5.902 | 3,30 ± 0,30 |  |  |  | Isobutylene epoxide (C_4_H_8_) |
| 5.919 |  |  |  | 3,58 ± 0,16 | 2-methyl-Propanal(C_4_H_8_O) |
| 6.820 |  |  |  | 1,15 ± 0,01 | 2,3-Butanedione(C_4_H_6_O_2_) |
| 8.080 | 1,55 ± 0,02 |  | 1,85 ± 0,00 | 2,27 ± 0,16 | 3-methyl-Butanal(C_5_H_10_O) |
| 8.282 | 1,43 ± 0,03 |  | 1,04 ± 0,03 | 1,62 ± 0,19 | 2-methyl-Butanal(C_5_H1_0_O) |
| 9.486 | 2,41 ± 0,11 |  |  | 5,95 ± 0,51 | Etanoic acid(CH_3_COOH) |
| 10.804 | 1,45 ± 0,08 | 0,84 ± 0,13 | 4,11 ± 0,02 | 7,40 ± 0,54 | 1-hydroxy-2-Propanone(C_3_H_6_O) |
| 11.055 | 4,18 ± 1,00 | 4,82 ± 0,81 | 5,37 ± 0,40 | 3,60 ± 0,12 | Toluene(C_7_H_8_) |
| 12.982 | 1,14 ± 0,04 | 2,45 ± 0,12 | 1,76 ± 0,18 |  | 3-methyl-Butanenitrile(C_5_H_9_N) |
| 15.555 |  |  | 0,89 ± 0,01 |  | (3,3-dimethylbutyl)- Benzene( C_12_H_18_) |
| 15.873 | 2,30 ± 0,09 | 2,93 ± 0,16 | 3,89 ± 0,29 | 3,78 ± 0,21 | Pyrrole (C_4_H_5_N) |
| 18.756 |  | 2,12 ± 0,05 |  |  | 4,4-Dimethyl-3-oxopentanenitrile (CH_11_NO) |
| 18.798 |  |  |  | 1,64 ± 0,03 | 2-Cyclopenten-1-one(C_5_H_6_O) |
| 19.161 | 1,41 ± 0,03 | 2,99 ± 0,05 | 1,86 ± 0,16 | 1,01 ± 0,06 | 4-methyl-Pentanenitrile(C_6_H_11_N) |
| 20.581 |  |  | 1,05 ± 0,12 | 0,91 ± 0,03 | 2-methyl-1H-Pyrrole(C_5_H_7_N) |
| 21.316 |  |  |  | 2,47 ± 0,03 | 2-Furanmethanol(C_5_H_6_O) |
| 21.385 |  | 2,82 ± 0,06 | 3,26 ± 0,16 |  | Acetamide(C_2_H_5_NO) |
| 21.552 |  |  |  | 1,98 ± 1,22 | 3-methyl-Butanoic acid (C_5_H_10_O) |
| 23.233 |  | 1,14 ± 0,01 |  |  | D-Limonene (C_10_H_16_) |
| 24.958 |  |  |  | 0,92 ± 0,11 | 2-hydroxy-  2-Cyclopenten-1-one(C_5_H_6_O) |
| 30.328 |  |  | 1,91 ± 0,17 | 2,4 ± 0,11 | 2-hydroxy-3-methyl-2-Cyclopenten-1-one (C_6_H_8_O_2_) |
| 32.151 | 4,31 ± 0,54 | 5,72 ± 0,21 | 4,97 ± 0,61 | 3,32 ± 0,02 | Phenol(C_6_H_6_O) |
| 36.409 |  | 1,81 ± 0,32 |  |  | 4-methyl-Phenol(C_21_H_28_O) |
| 34.877 |  |  |  | 0,85 ± 0,04 | 2-hydroxy-3-ethyl-2-Cyclopenten-  1-one(C_7_H_10_O) |
| 36.413 | 2,16 ± 0,45 |  | 2,15 ± 0,33 | 1,73 ± 0,02 | 4-methyl-Phenol(C_21_H_28_O) |
| 37.977 | 1,00 ± 0,08 | 1,49 ± 0,08 | 0,94 ± 0,08 |  | Benzyl nitrile(C_8_H_7_N) |
| 38.384 |  |  |  | 1,53 ± 0,13 | Cyclopropyl carbinol(C_4_H_8_O) |
| 39.002 | 1,09 ± 0,10 | 1,35 ± 0,04 | 1,20 ± 0,11 | 0,97 ± 0,06 | Hexadecanamide(C_16_H_33_NO) |
| 40.652 | 1,12 ± 0,21 |  |  |  | 9-Oxabicyclo[4.2.1]nonane  (C_8_H_14_O) |
| 43.527 | 1,52 ± 0,03 | 2,43 ± 010 | 1,92 ± 0,08 | 1,39 ± 0,13 | Benzenepropanenitrile  (C_9_H_9_N) |
| 43.756 |  |  |  | 0,92 ± 0,01 | 1,4:3,6-Dianhydro-.alpha.-d-glucopyranose (C_6_H_8_O_4_) |
| 47.545 | 3,87 ± 0,30 | 5,62 ± 0,17 | 4,86 ± 0,49 | 3,13 ± 0,05 | Indole (C_8_H_7_N) |
| 49.058 | 1,14 ± 0,14 |  | 1,04 ± 0,02 |  | 2,4-Dimethyl-2-oxazoline-4-methanol  (C_6_H_11_NO_2_) |
| 50.805 |  |  | 0,89 ± 0,02 | 0,96 ± 0,05 | 1H-Indole, 3-methyl- |
| 51.503 |  | 1,13 ± 0,13 |  |  | 1,4-Cyclohexanedione (C_6_H_8_O_2_) |
| 53.090 | 6,41 ± 0,03 | 3,11 ± 0,66 | 2,84 ± 0,23 | 6,95 ± 0,54 | Heptadecane (C_17_H_36_) |
| 53.300 | 1,41 ± 0,05 |  |  | 0,96 ± 0,07 | 2,4-Dimethyl-2-oxazoline-4-methanol (C_6_H_11_NO) |
| 55.546 | 1,80 ± 0,04 | 2,65 ± 0,04 | 1,88 ± 0,02 | 1,17 ± 0,07 | 1-(cyanoacetyl)-Piperidine  (C_8_H_12_N_2_O) |
| 56.371 |  | 0,93 ± 0,04 |  |  | 1-Azepan-1-yl-2-bromethanone (C_12_H_23_NO) |
| 58.392 | 0,90 ± 0,06 | 1,18 ± 0,04 | 1,15 ± 0,03 |  | 3,7,11,15-tetramethyl-2-Hexadecene (C_20_H_40_) |
| 58.796 | 0,99 ± 0,02 | 0,96 ± 0,08 |  |  | 3,7,11,15-tetramethyl-2-Hexadecene isomer (C_20_H_40_) |
| 59.166 | 7,52 ± 0,18 | 5,78 ± 0,54 | 5,77 ± 0,13 | 5,31 ± 0,08 | 3,7,11,15-Tetramethyl-2-hexadecen-1-ol (C_20_H_40_O) |
| 59.355 | 0,97 ± 0,09 |  |  |  | 1-(5-methyl-2-propan-2-ylcyclohexyl)oxy-3-morpholin-4-yl-propan-2-ol  (C_17_H_33_NO_3_) |
| 60.045 | 1,63 ± 0,06 |  |  |  | Palmitoyl chloride (C_l1_6H_31_ClO) |
| 60.145 |  | 1,20 ± 0,31 |  |  | N,N-dimethyl-3-Octen-2-amine  (C_10_H_12_N) |
| 60.629 | 1,54 ± 0,04 | 1,14 ± 0,01 | 1,01 ± 0,18 | 0,89 ± 0,01 | m-Menth-1(7)-ene(C_10_H_18_) |
| 60.806 | 2,55 ± 0,13 | 1,95 ± 0,02 | 1,94 ± 0,13 | 1,67 ± 0,05 | Phytol (C_20_H_40_O) |
| 63.165 |  | 0,90 ± 0,10 |  |  | 3-Ethyl-5-methylindolizidine (C_11_H_21_N) |
| 64.130 |  |  | 1,90 ± 1,44 |  | 2-Ethyl-3-methoxypyrazine (C_7_H_10_N_2_O) |
| 64.139 | 1,73 ± 0,01 |  |  |  | 3-methoxy-2-methyl-Phenol (C_8_H_10_O_2_) |
| 66.232 |  | 1,63 ± 0,21 |  |  | 3-(3,4,5-trimethoxyphenyl) Acrylic acid (C_22_H_31_NO_5_) |
| 66.232 | 0,95 ± 0,01 |  | 1,35 ± 0,21 |  | Bis(2-methylpropyl)-Phthalate (C_16_H_22_O_4_) |
| 66.606 | 4,61 ± 0,98 | 7,06 ± 1,01 | 3,75 ± 0,49 | 3,10 ± 0,36 | Heptadecanenitrile (C_17_H_33_N) |
| 68.809 |  |  | 7,54 ± 0,57 | 12,60 ± 0,19 | Pentadecanoic acid (C_15_H_30_O) |
| 68.901 | 15,23 ± 0,39 |  |  |  | l-(+)-Ascorbic acid 2,6-dihexadecanoate (C_38_H_68_O_8_) |
| 71.296 |  |  | 1,16 ± 0,34 |  | E-Phytol (C_20_H_40_O) |
| 72.777 |  | 1,72 ± 0,02 | 0,85 ± 0,13 |  | Oleanitrile (C_18_H_33_N) |
| 73.059 | 1,37 ± 0,52 |  |  |  | 9-Octadecyne (C_18_H_34_) |
| 77.826 | 7,01 ± 1,64 | 10,72 ± 1,00 | 8,16 ± 0,91 | 4,23 ± 0,06 | Hexadecanamide (C_16_H_33_NO) |

*Retention Time (RT),, ± standard error.

**S3 Table List of semi-quantified compounds (% area) from pyrolysis of A, M2.5, M5, M10 at 650ºC.**

|  | **A** | **M2.5** | **M5** | **M10** | **Compounds** |
| --- | --- | --- | --- | --- | --- |
| **RT** | **Concentration (% area)** | | | | **Name** |
| 5.809 | 7,18 ± 0,43 | 9,77 ± 0,30 |  |  | Acetonitrila (C₂H₃N) |
| 5.880 |  |  |  | 7,41 ± 0,64 | Isobutylene epoxide (C_4_H_8_) |
| 6.784 |  |  |  | 1,41 ± 0,04 | 2,3-Butanedione (C_4_H_6_O_2_) |
| 7.469 | 1,10 ± 0,03 | 2,09 ± 0,19 |  |  | Propanenitrile (C_3_H_5_N) |
| 8.072 | 1,70 ± 0,08 |  | 2,05 ± 0,16 | 2,49 ± 0,10 | 3-methyl-Butanal(C_5_H_10_O) |
| 8.248 | 1,37 ± 0,10 |  | 1,13 ± 0,12 | 1,78 ± 0,10 | 2-methyl-Butanal(C_5_H1_0_O) |
| 9.464 | 2,01 ± 0,69 |  |  |  | Ammonium acetate (C_2_H_7_NO_2_) |
| 10.572 |  | 0,91 ± 0,02 |  |  | Butanenitrile (C_4_H_7_N) |
| 10.823 | 1,47 ± 0,09 |  | 3,48 ± 0,93 | 7,04 ± 0,06 | 1-hydroxy-2-Propanone(C_3_H_6_O) |
| 11.027 | 7,79 ± 0,26 | 8,06 ± 0,52 | 7,50 ± 0,68 | 7,49 ± 0,01 | Toluene(C_7_H_8_) |
| 12.947 | 1,98 ± 0,00 | 2,96 ± 0,11 | 2,26 ± 0,01 | 1,73 ± 0,05 | 3-methyl-Butanenitrile(C_5_H_9_N) |
| 15.506 |  |  |  | 1,42 ± 0,04 | 1,7-Octadiyne (C_8_H_10_) |
| 15.530 | 1,14 ± 0,04 | 1,33 ± 0,02 | 1,39 ± 0,03 |  | Ethylbenzene (C_8_H_10_) |
| 15.834 | 2,24 ± 0,03 | 2,73 ± 0,01 | 4,01 ± 1,05 | 5,27 ± 0,12 | Pyrrole (C_4_H_5_N) |
| 17.943 | 0,99 ± 0,08 |  |  | 1,05 ± 0,25 | Styrene (C_8_H_8_) |
| 18.736 |  |  |  | 1,59 ± 0,77 | 2-Cyclopenten-1-one(C_5_H_6_O) |
| 18.753 |  | 2,10 ± 0,04 |  |  | 4,4-Dimethyl-3-oxopentanenitrile (CH_11_NO) |
| 19.113 | 1,83 ± 0,06 | 3,24 ± 0,04 | 2,14 ± 0,07 | 1,69 ± 0,03 | 4-methyl-Pentanenitrile(C_6_H_11_N) |
| 20.201 | 1,03 ± 0,02 |  |  | 1,02 ± 0,14 | 2-methyl-1H-Pyrrole (C_5_H_7_N) |
| 20.535 | 0,76 ± 0,06 | 0,98 ± 0,04 | 1,26 ± 0,03 | 1,47 ± 0,11 | [Alpha-Methylpyrrole](https://www.ncbi.nlm.nih.gov/pcsubstance/?term=%22alpha-Methylpyrrole%22%5bCompleteSynonym%5d%20AND%2012489%5bStandardizedCID%5d) (C_5_H_7_N) |
| 21.282 |  |  |  | 2,07 ± 0,44 | 2-Furanmethanol(C_5_H_6_O) |
| 21.421 |  | 1,82 ± 0,27 | 3,60 ± 0,49 |  | Acetamide(C_2_H_5_NO) |
| 23.234 | 0,8 ± 0,16 | 1,14 ± 0,28 |  |  | D-Limonene (C_10_H_16_) |
| 25.374 |  | 0,87 ± 0,18 | 0,91 ± 0,16 |  | 2,4-dimethyl-1H-Pyrrole (C_6_H_9_N) |
| 25.643 | 1,22 ± 0,08 |  |  |  | 2-Propenoic acid octyl ester (C_11_H_20_O) |
| 30.317 |  |  |  | 2,52 ± 0,15 | 2-hydroxy-3-methyl-2-Cyclopenten-1-one (C_6_H_8_O_2_) |
| 30.340 | 0,99 ± 0,22 |  |  |  | 3-Heptyn-1-ol (C_7_H_20_O) |
| 32.124 | 5,00 ± 0,28 | 5,58 ± 0,32 | 4,90 ± 0,05 | 4,06 ± 0,07 | Phenol(C_6_H_6_O) |
| 34.602 |  | 0,88 ± 0,06 |  |  | 2-methyl-Phenol(C_21_H_28_O) |
| 35.771 |  | 0,87 ± 0,18 |  |  | Tridecane (C_13_H_28_) |
| 36.425 | 3,4 ± 0,22 | 2,66 ± 0,05 | 2,41 ± 0,03 | 2,36 ± 0,18 | 4-methyl-Phenol(C_21_H_28_O) |
| 37.897 | 1,27 ± 0,02 | 1,48 ± 0,06 | 0,87 ± 0,09 | 0,77 ± 0,00 | Benzyl nitrile(C_8_H_7_N) |
| 38.980 | 0,84 ± 0,01 | 0,89 ± 0,03 | 0,88 ± 0,05 | 0,91 ± 0,08 | Hexadecanamide(C_16_H_33_NO) |
| 40.404 |  | 1,51 ± 0,07 |  | 0,74 ± 0,03 | Tetradecane(C_14_H_30_) |
| 40.648 | 2,23 ± 0,06 | 2,22 ± 0,07 |  |  | 1-Tridecene (C_13_H_26_) |
| 40.651 |  |  | 0,86 ± 0,28 |  | 1-Tetradecene (C_14_H_28_) |
| 43.487 | 1,62 ± 0,06 | 2,19 ± 0,13 | 1,70 ± 0,06 | 1,51 ± 0,13 | Benzenepropanenitrile(C_9_H_9_N) |
| 44.848 | 0,77 ± 0,23 | 2,23 ± 0,23 | 1,45 ± 0,47 | 0,70 ± 0,01 | Pentadecane(C_15_H_32_) |
| 45.102 |  | 1,82 ± 0,33 | 1,16 ± 0,52 |  | 1-Pentadecene (C_15_H_30_) |
| 47.511 | 3,76 ± 0,09 | 5,32 ± 0,28 | 4,42 ± 0,09 | 3,35 ± 0,41 | Indole (C_8_H_7_N) |
| 50.766 | 1,32 ± 0,04 |  | 0,88 ± 0,01 | 1,20 ± 0,04 | 1H-Indole, 3-methyl- |
| 53.055 | 5,35 ± 0,18 | 2,60 ± 0,99 | 2,64 ± 0,21 | 6,41 ± 0,92 | Heptadecane (C_17_H_36_) |
| 58.392 | 0,90 ± 0,06 | 0,85 ± 0,10 | 0,81 ± 0,18 |  | 3,7,11,15-tetramethyl-2-Hexadecene (C_20_H_40_) |
| 58.793 | 1,02 ± 0,03 | 0,92 ± 0,01 | 0,77 ± 0,13 |  | 3,7,11,15-tetramethyl-2-Hexadecene isomer (C_20_H_40_) |
| 59.073 | 7,20 ± 0,00 | 5,09 ± 0,03 | 5,79 ± 0,48 | 5,01 ± 0,51 | 3,7,11,15-Tetramethyl-2-hexadecen-1-ol (C_20_H_40_O) |
| 60.706 | 0,92 ± 0,13 |  |  |  | m-Menth-1(7)-ene(C_10_H_18_) |
| 60.760 | 1,93 ± 0,23 | 1,14 ± 0,13 |  | 1,24 ± 0,11 | Phytol (C_20_H_40_O) |
| 66.491 | 6,02 ± 0,78 | 7,09 ± 0,04 | 4,14 ± 1,22 | 3,43 ± 0,30 | Heptadecanenitrile (C_17_H_33_N) |
| 68.751 | 8,90 ± 0,75 |  | 5,96 ± 2,97 | 6,16 ± 0,91 | Pentadecanoic acid (C_15_H_30_O_2_) |
| 72.774 | 0,98 ± 0,17 | 1,31 ± 0,09 |  |  | Oleanitrile (C_18_H_33_N) |
| 77.760 | 6,86 ± 2,47 | 8,78 ± 2,03 | 8,00 ± 1,28 | 1,52 ± 0,08 | Hexadecanamide (C_16_H_33_NO) |

*Retention Time (RT),, ± standard error.

**S4 Table – Group of the volatile compounds (oxygenated, nitrogenates, N and O containing compounds, phenols, non-aromatic hydrocarbons and aromatic hydrocarbons) obtained by pyrolysis. The tukey test was applied with a significance level of 5% to evaluate of process temperature.**

|  | **450 °C** | **550 °C** | **650 °C** |
| --- | --- | --- | --- |
|  | **A** | | |
| **Compounds Group** | **Yields (%area)** | | |
| Oxigenated | ^a^51.7±0.4 | ^b^43.6±0.9 | ^c^28.0±1.6 |
| Nitrogenates | ^c^13.9±1.0 | ^b^18.2±2.1 | ^a^32.4±0.0 |
| N/O | ^b^9.93±0.8 | a13.5±1.6 | ^c^8.0±2.4 |
| Phenolics | ^c^5.29±0.5 | ^b^8.9±1.0 | ^a^8.8±0.7 |
| HC | ^a^19.2±1.2 | ^c^11.3±0.8 | ^b^11.7±0.0 |
| Aromatic HC | ^b^ND | ^b^4.5±1.0 | ^a^11.2±0.2 |
|  | **M2.5** | | |
| Oxigenated | ^a^20.6±0.2 | ^b^14.0±0.3 | ^c^6.68±0.3 |
| Nitrogenates | ^c^23.5±2.2 | ^b^32.7±0.3 | ^a^42.8±1.3 |
| N/O | ^a^39.0±1.0 | ^b^28.6±0.6 | ^c^15.5±2.2 |
| Phenolics | ^c^7.90±0.2 | ^b^9.35±0.8 | ^a^9.76±0.1 |
| HC | ^c^9.01±1.2 | ^b^9.33±0.6 | ^a^15,2±1.0 |
| Aromatic HC | ^c^ND | ^b^6.00±1.1 | ^a^10.0±0.3 |
|  | **M5** | | |
| Oxigenated | ^a^37.2±1.5 | ^b^29.4±0.8 | ^c^23.7±2.9 |
| Nitrogenates | ^c^21.0±0.1 | ^a^31.0±1.1 | ^b^29.2±1.7 |
| N/O | ^a^27.2±1.6 | ^b^19.3±2.6 | ^c^16.1±1.6 |
| Phenolics | ^c^4.4±0.7 | ^a^7.8±0.9 | ^b^9.5±0.4 |
| HC | ^a^10.3±0.7 | ^c^5.5±0.6 | ^b^10.0±2.8 |
| Aromatic HC | ^c^ND | ^b^6.9±0.3 | ^b^11.5±0.3 |
|  | **M10** | | |
| Oxigenated | ^a^75.4±2.5 | ^a^b58.8±0.5 | ^c^44.6±2.5 |
| Nitrogenates | ^c^7.8±0.5 | ^b^13.6±2.7 | ^a^24.7±1.3 |
| N/O | ^a^12.2±1.8 | ^b^9.7±2.5 | ^c^2.8±0.1 |
| Phenolics | ^c^1.7±0.4 | ^b^5.5±0.0 | ^a^7.4±0.9 |
| HC | ^c^2.8±0.2 | ^b^8.5±0.6 | ^a^10.7±0.0 |
| Aromatic HC | ^b^ND | ^b^3.9±0.1 | ^a^9.8±0.6 |

Tukey Test (Different letters indicate significant differences for different treatments (p < 0.05).


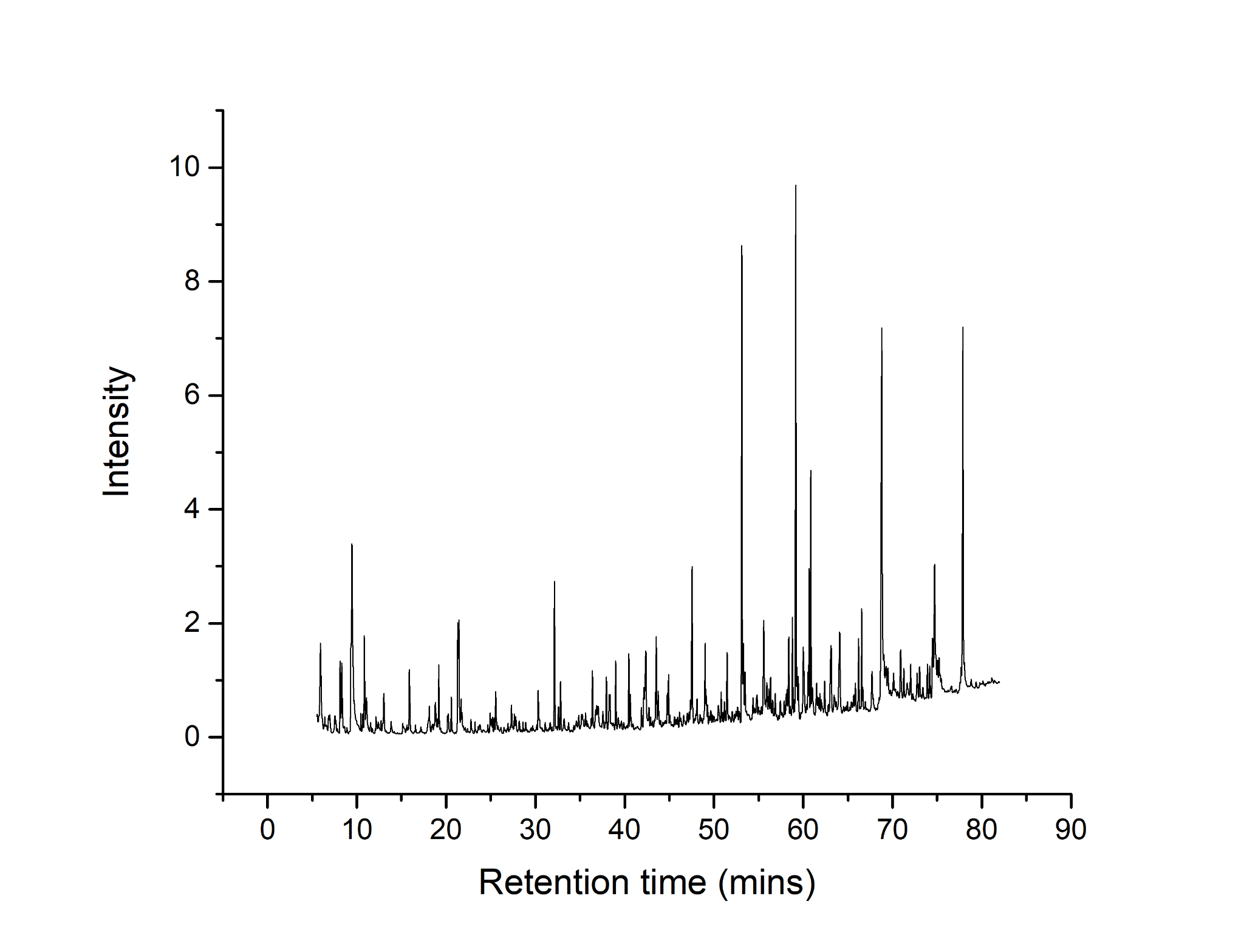


**S1 Fig - Pyrograms of volatile compounds analysis by GC/MS from pyrolysis of sample A at 450°C (1)**


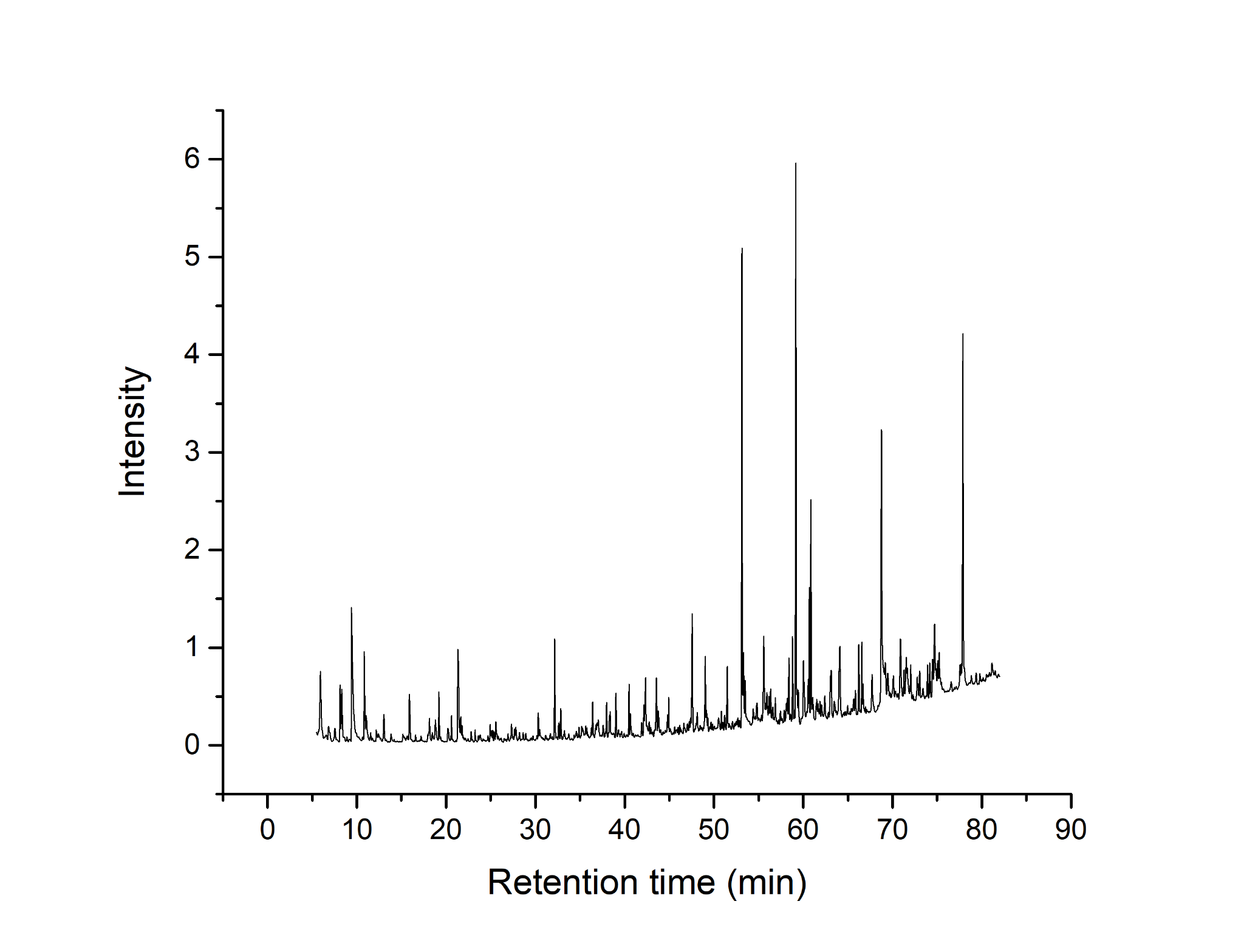


**S2 Fig - Pyrograms of volatile compounds analysis by GC/MS from pyrolysis of sample A at 450°C (2).**


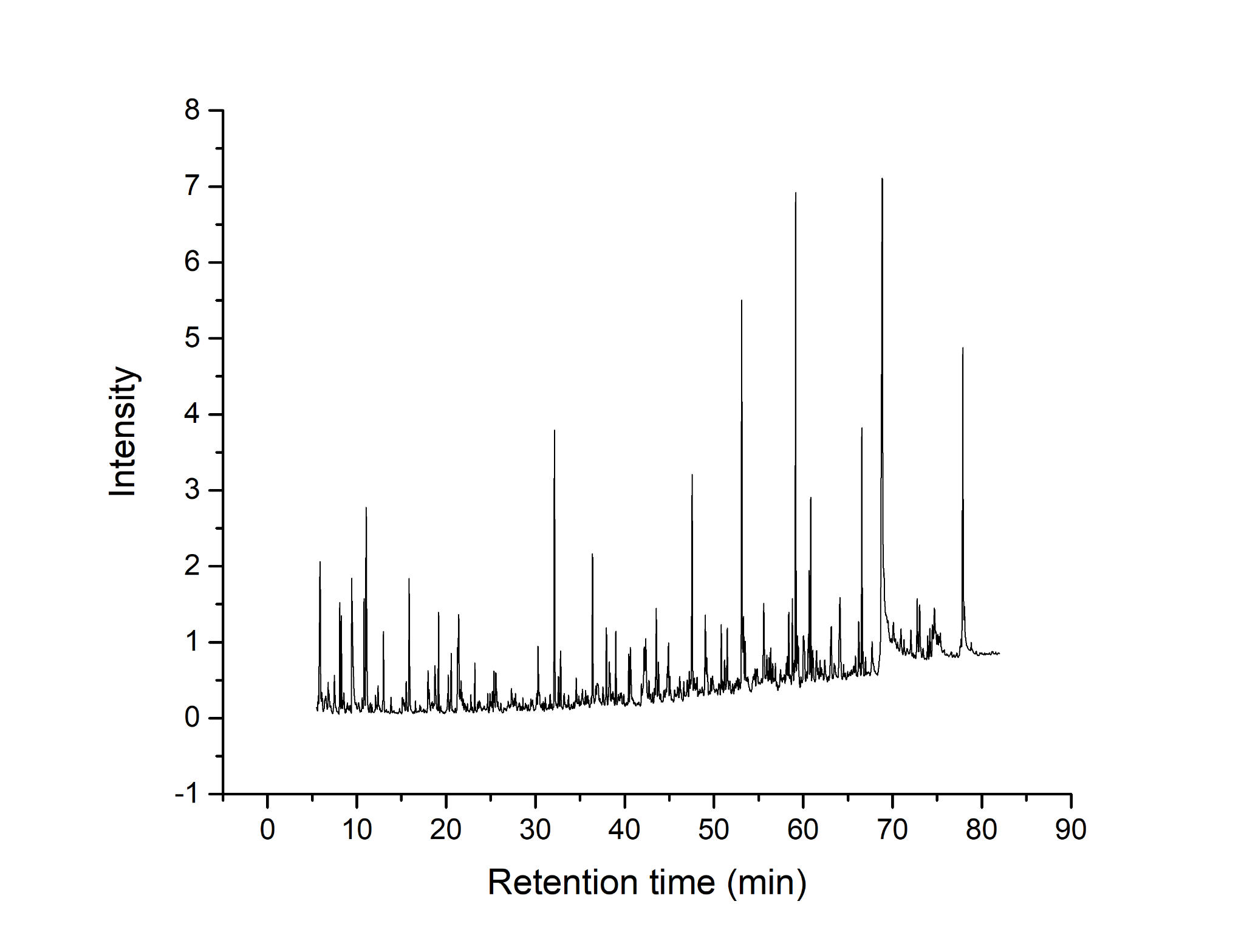


**S3 Fig - Pyrograms of volatile compounds analysis by GC/MS from pyrolysis of sample A at 550°C (1).**

**
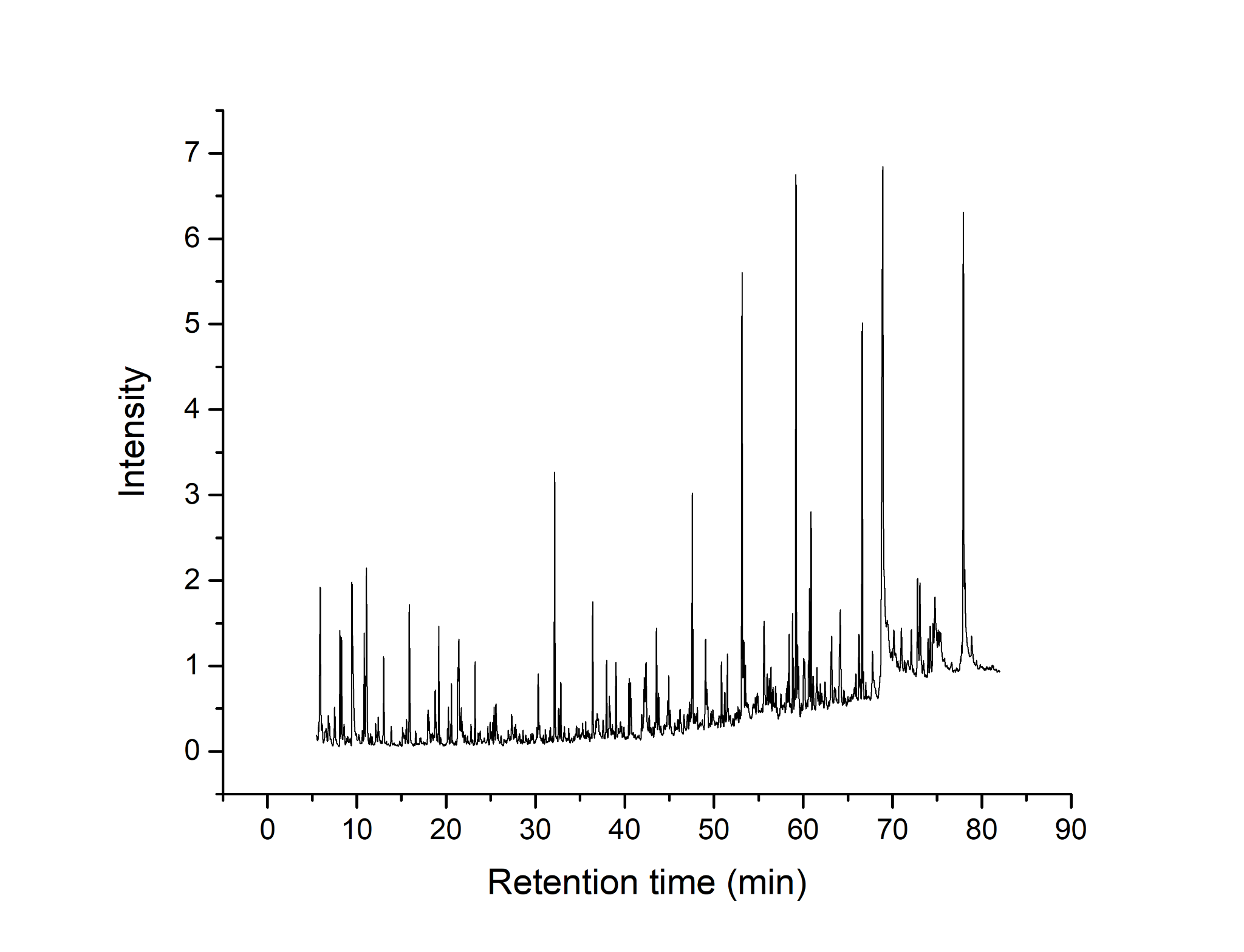
**

**S4 Fig - Pyrograms of volatile compounds analysis by GC/MS from pyrolysis of sample A at 550°C (2).**

**
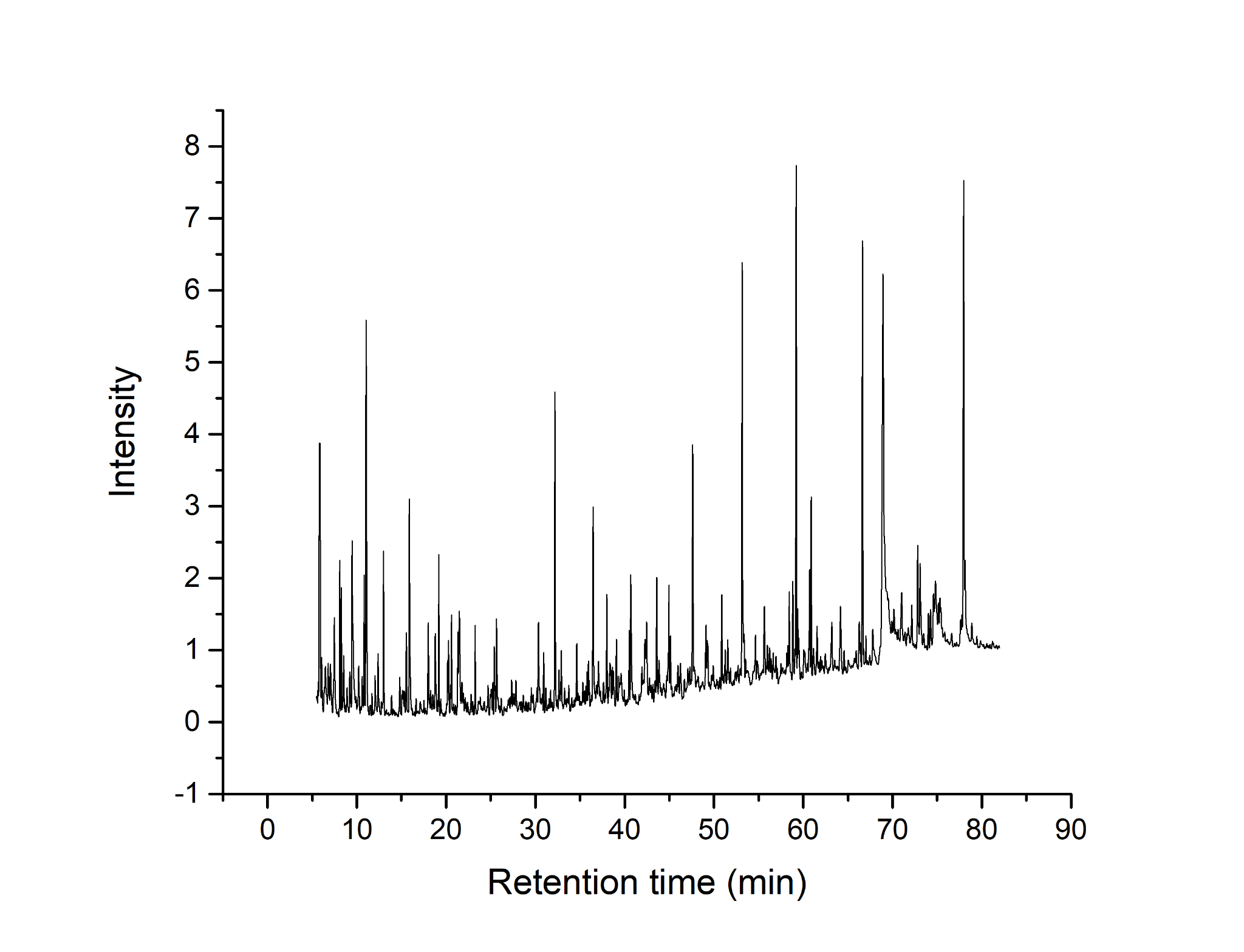
**

**S5 Fig - Pyrograms of volatile compounds analysis by GC/MS from pyrolysis of sample A at 650°C (1).**

**
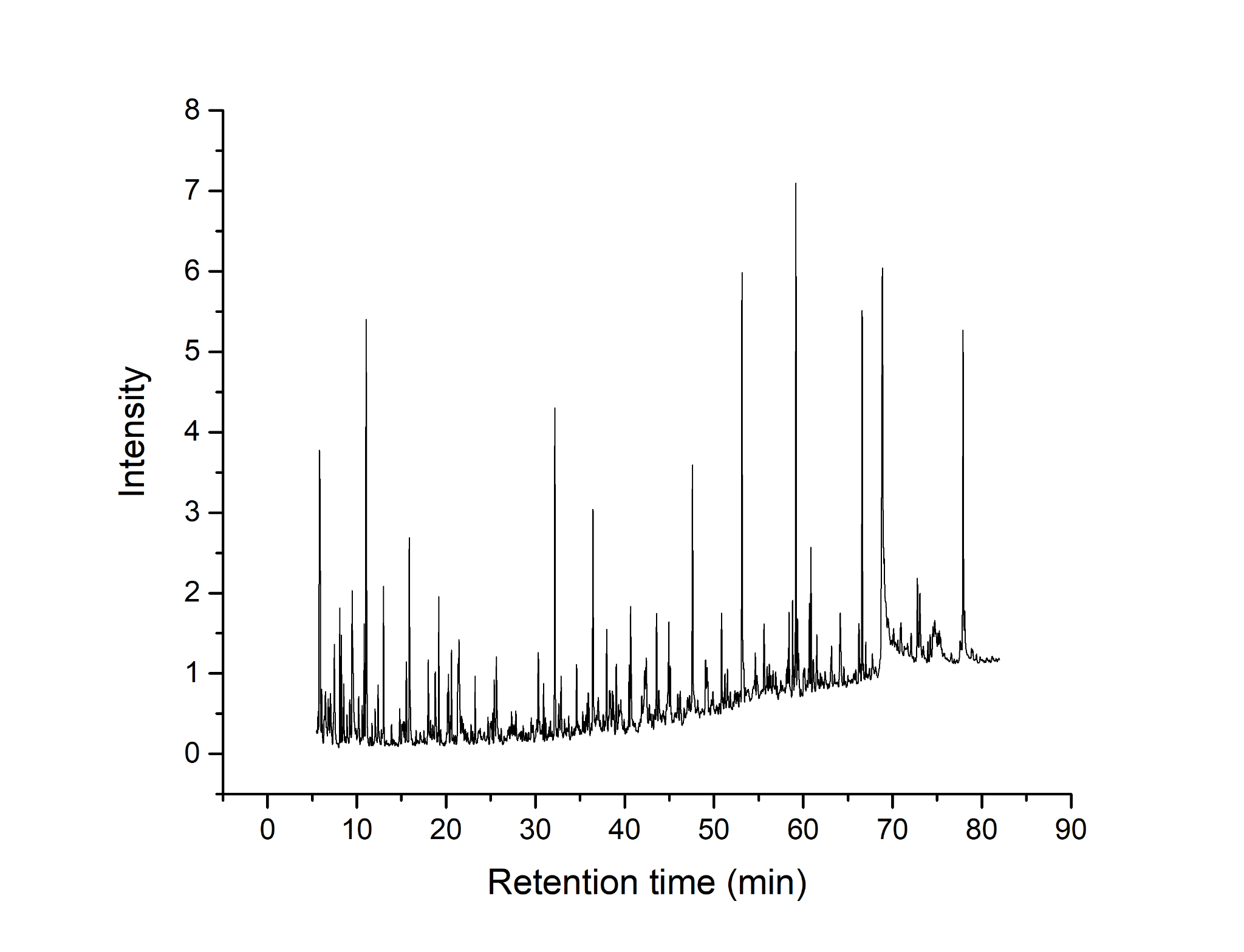
**

**S6 Fig - Pyrograms of volatile compounds analysis by GC/MS from pyrolysis of sample A at 650°C (2).**

**
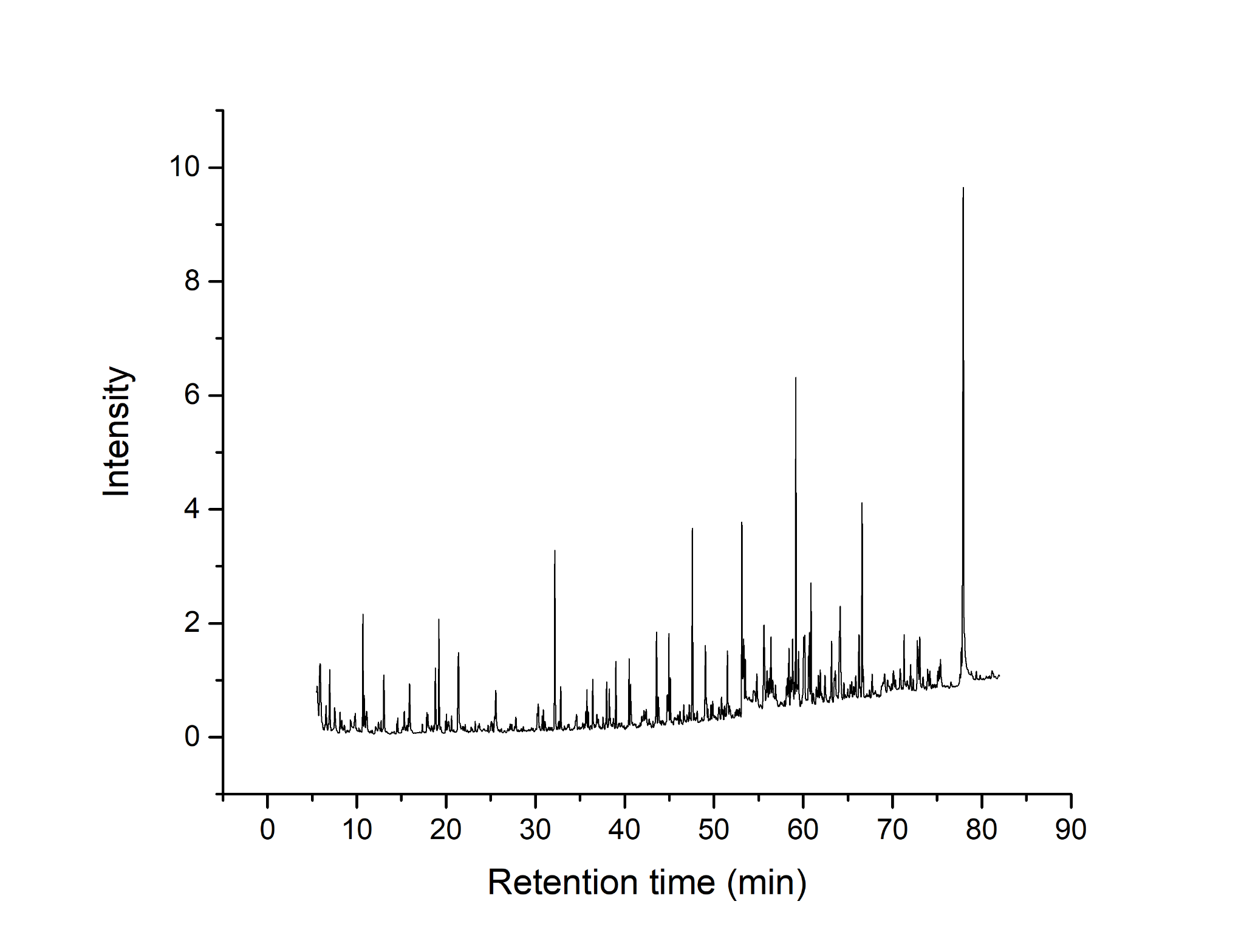
**

**S7 Fig - Pyrograms of volatile compounds analysis by GC/MS from pyrolysis of sample M2.5 at 450°C (1).**

**
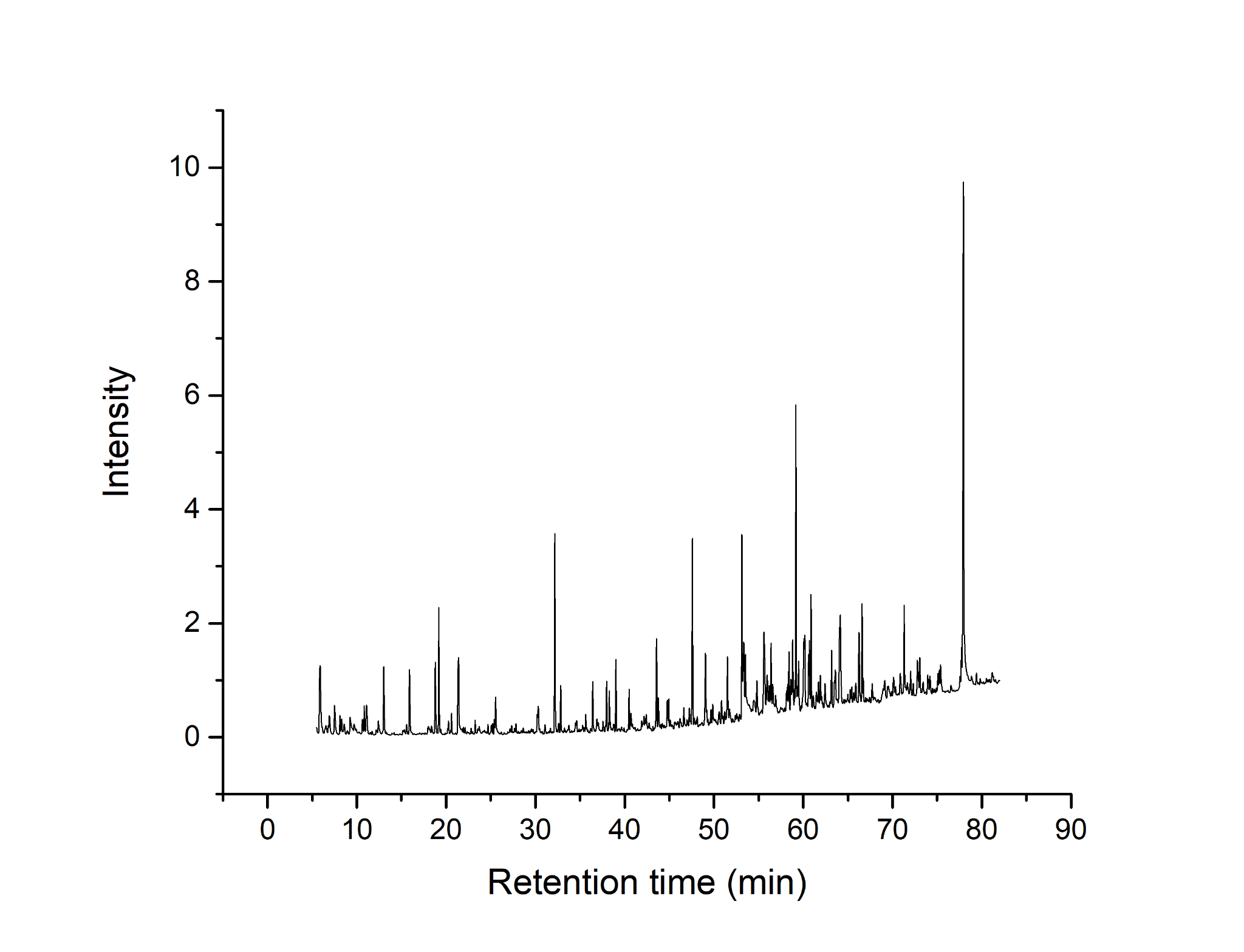
**

**S8 Fig - Pyrograms of volatile compounds analysis by GC/MS from pyrolysis of sample M2.5 at 450°C (2).**

**
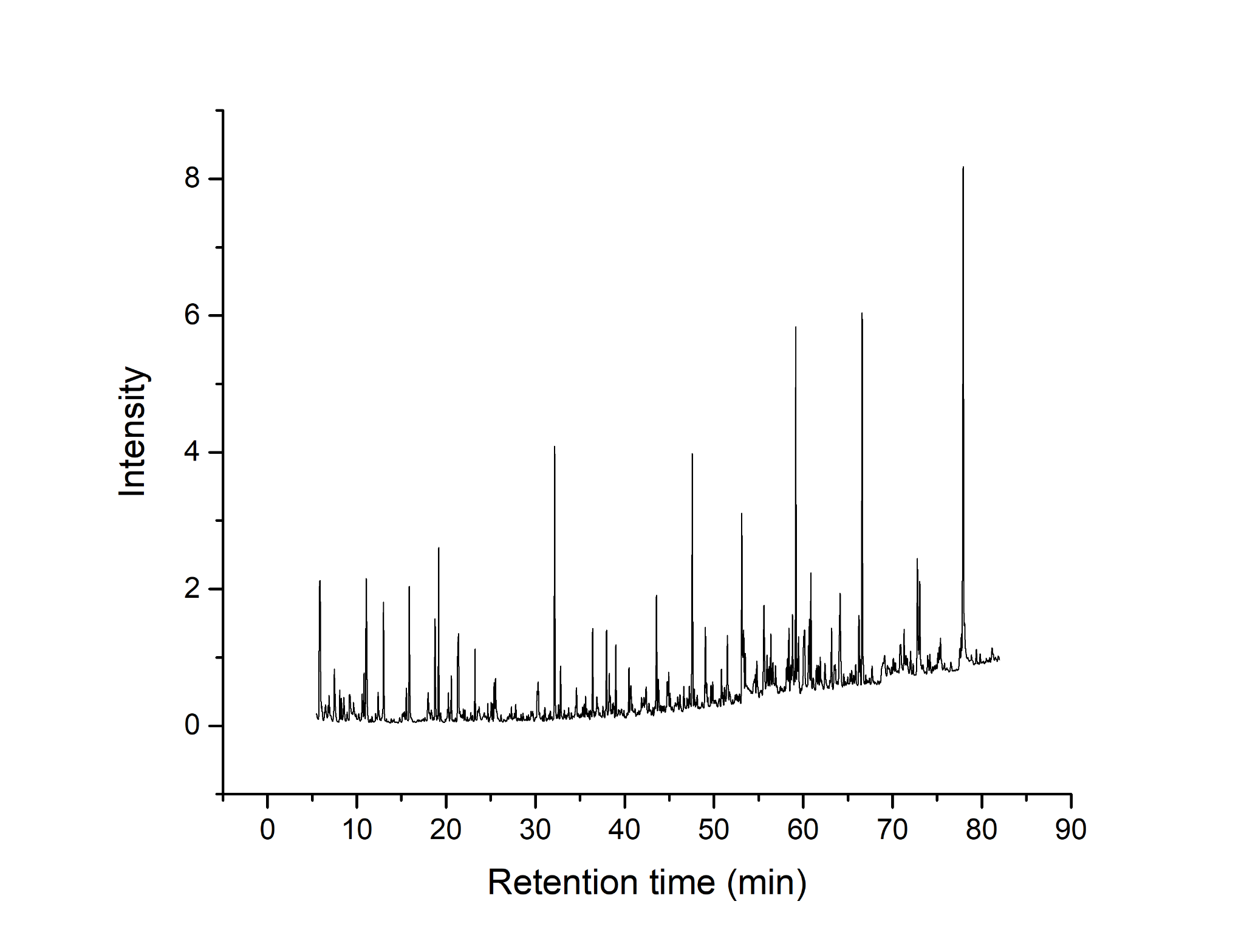
**

**S9 Fig - Pyrograms of volatile compounds analysis by GC/MS from pyrolysis of sample M2.5 at 550°C (1).**


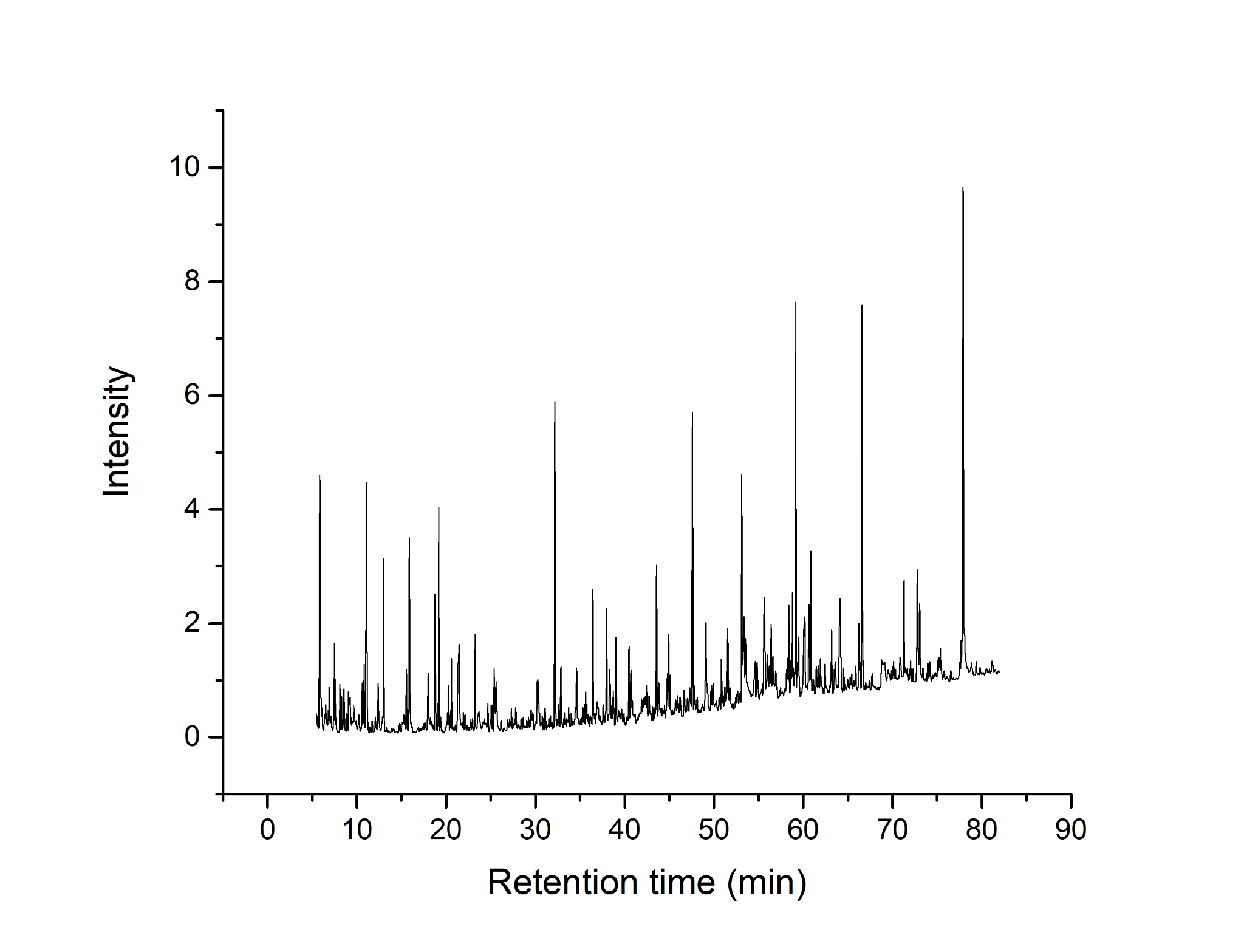


**S10 Fig - Pyrograms of volatile compounds analysis by GC/MS from pyrolysis of sample M2.5 at 550°C (2).**

**
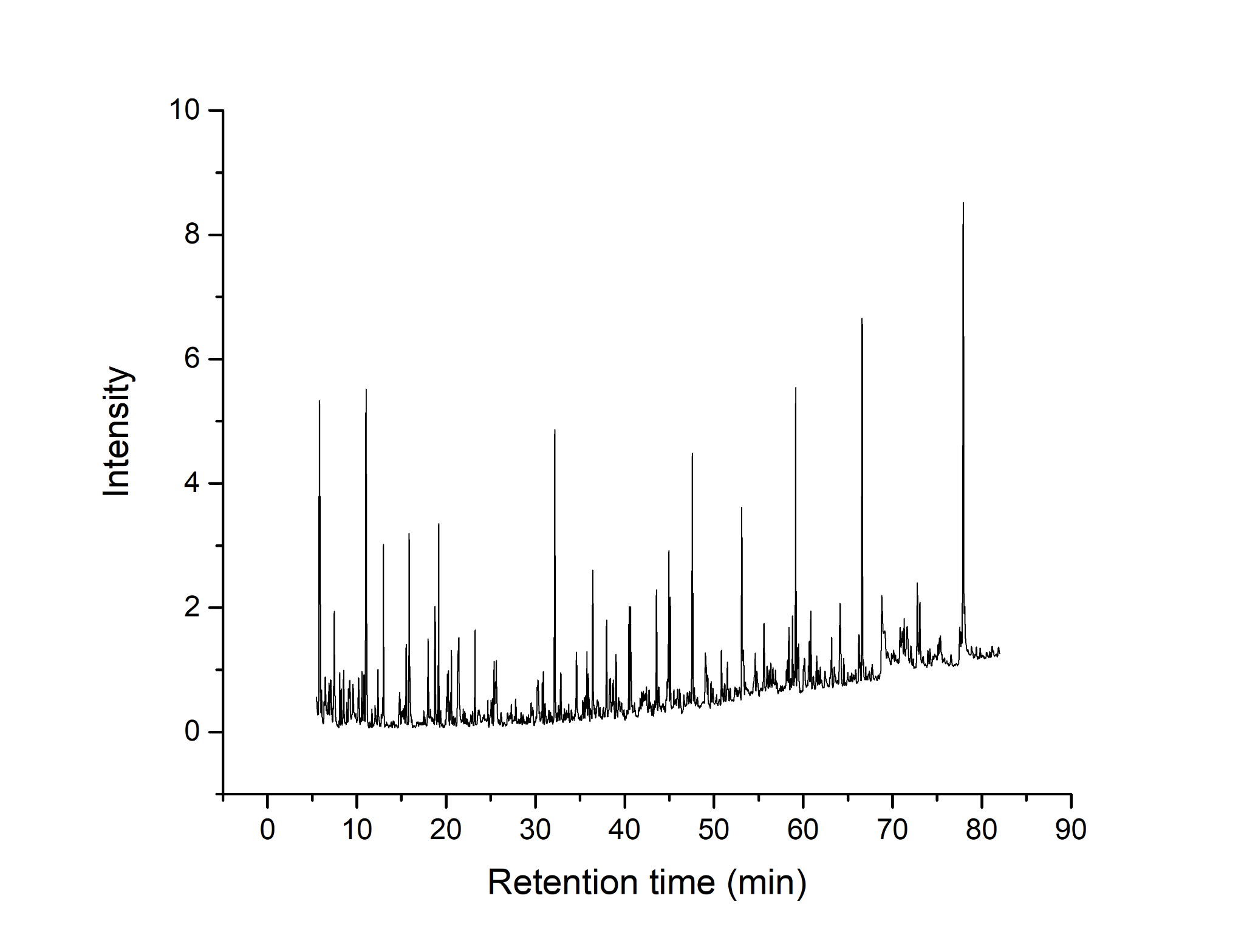
**

**S11 Fig - Pyrograms of volatile compounds analysis by GC/MS from pyrolysis of sample M2.5 at 650°C (1).**

**
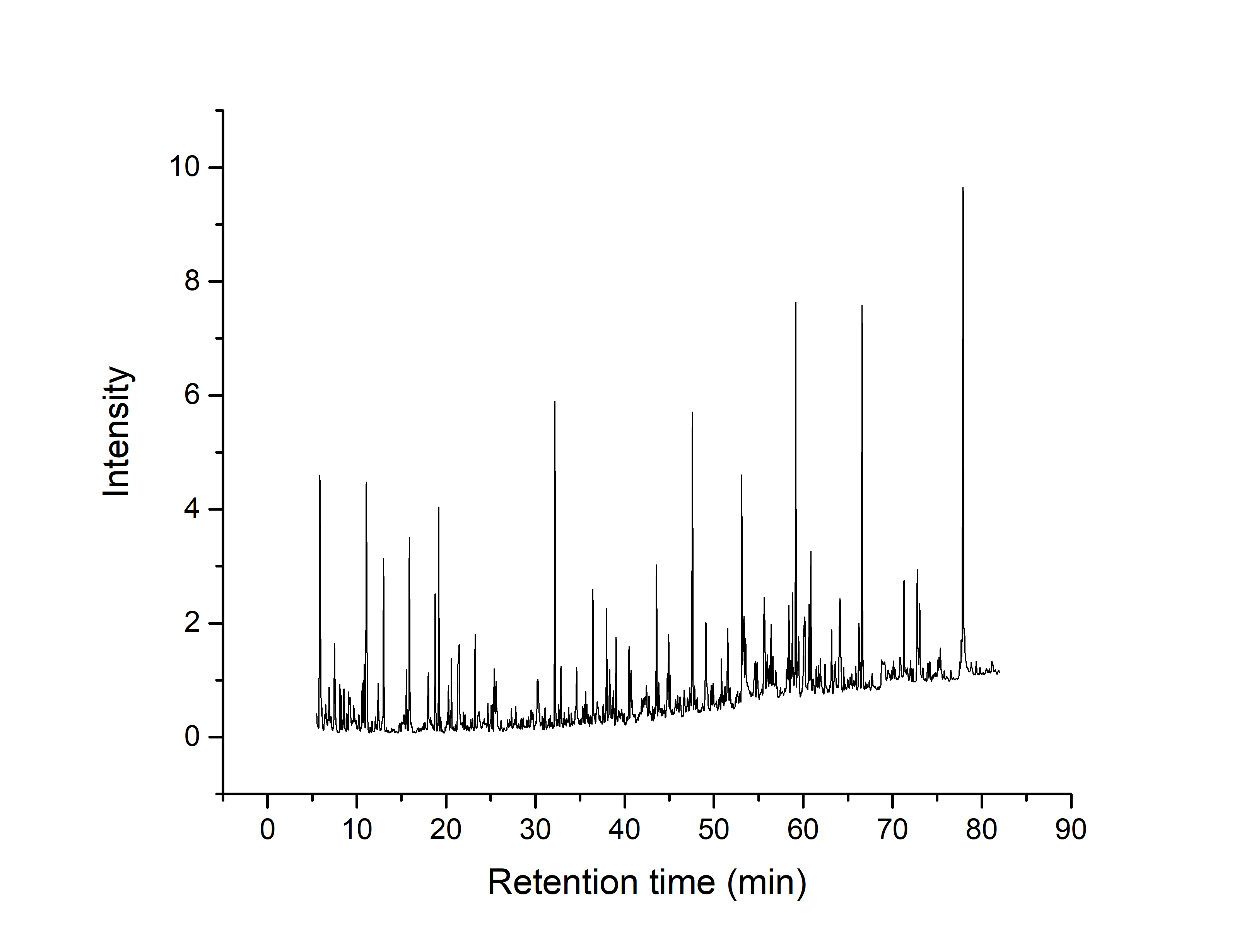
**

**S12 Fig - Pyrograms of volatile compounds analysis by GC/MS from pyrolysis of sample M2.5 at 650°C (2).**

**
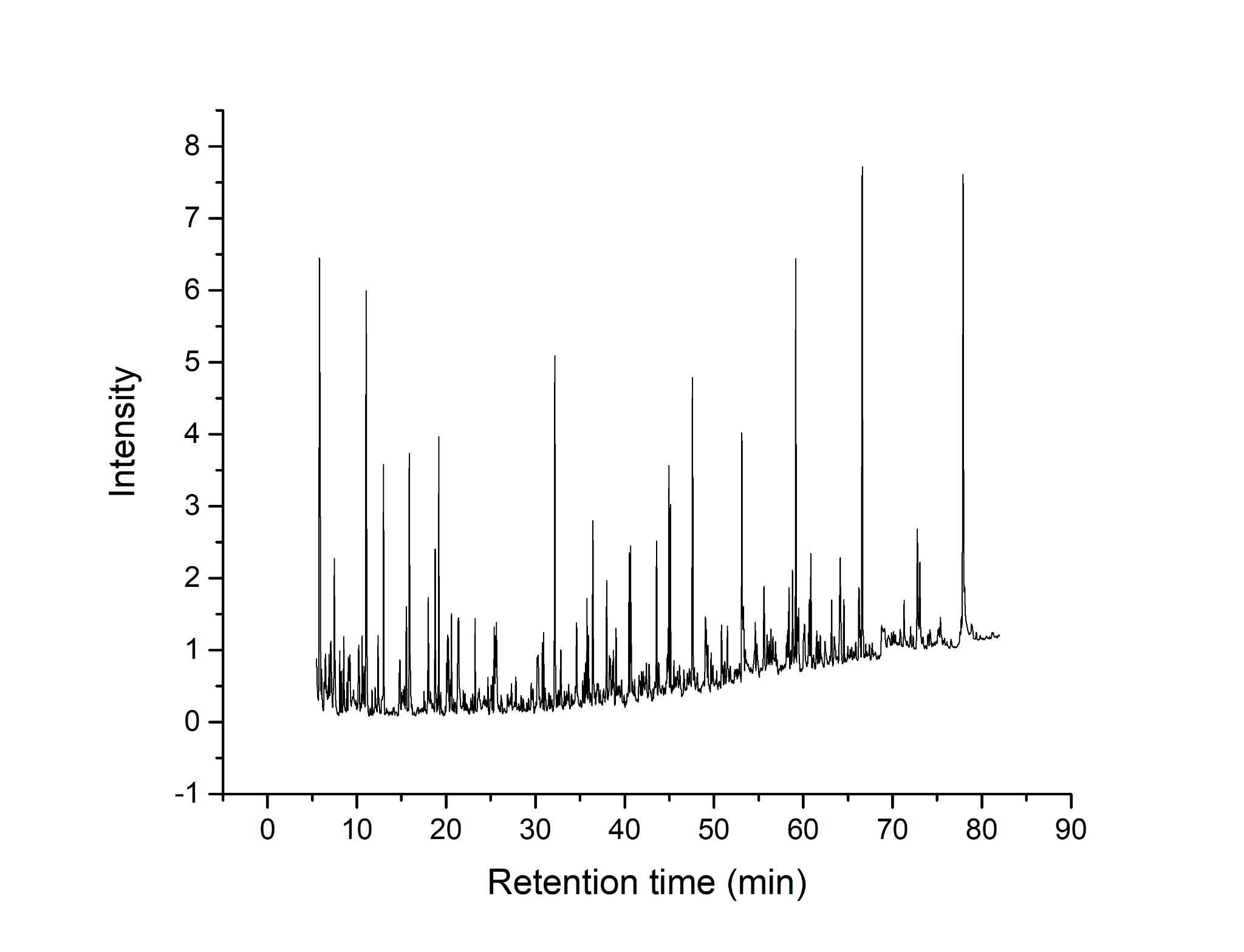
**

**S13 Fig - Pyrograms of volatile compounds analysis by GC/MS from pyrolysis of sample M5 at 450°C (1).**

**
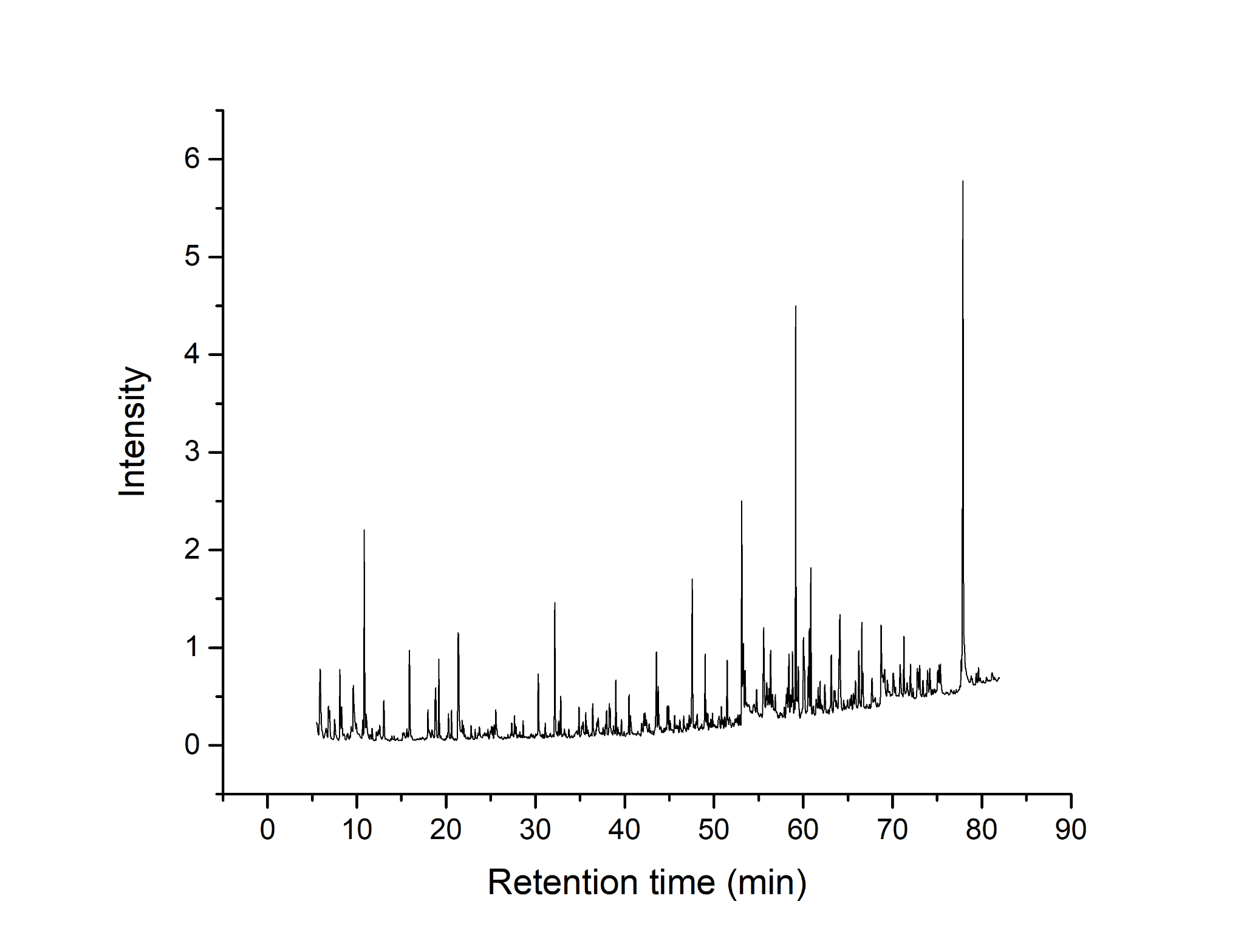
**

**S14 Fig - Pyrograms of volatile compounds analysis by GC/MS from pyrolysis of sample M5 at 450°C (2).**

**
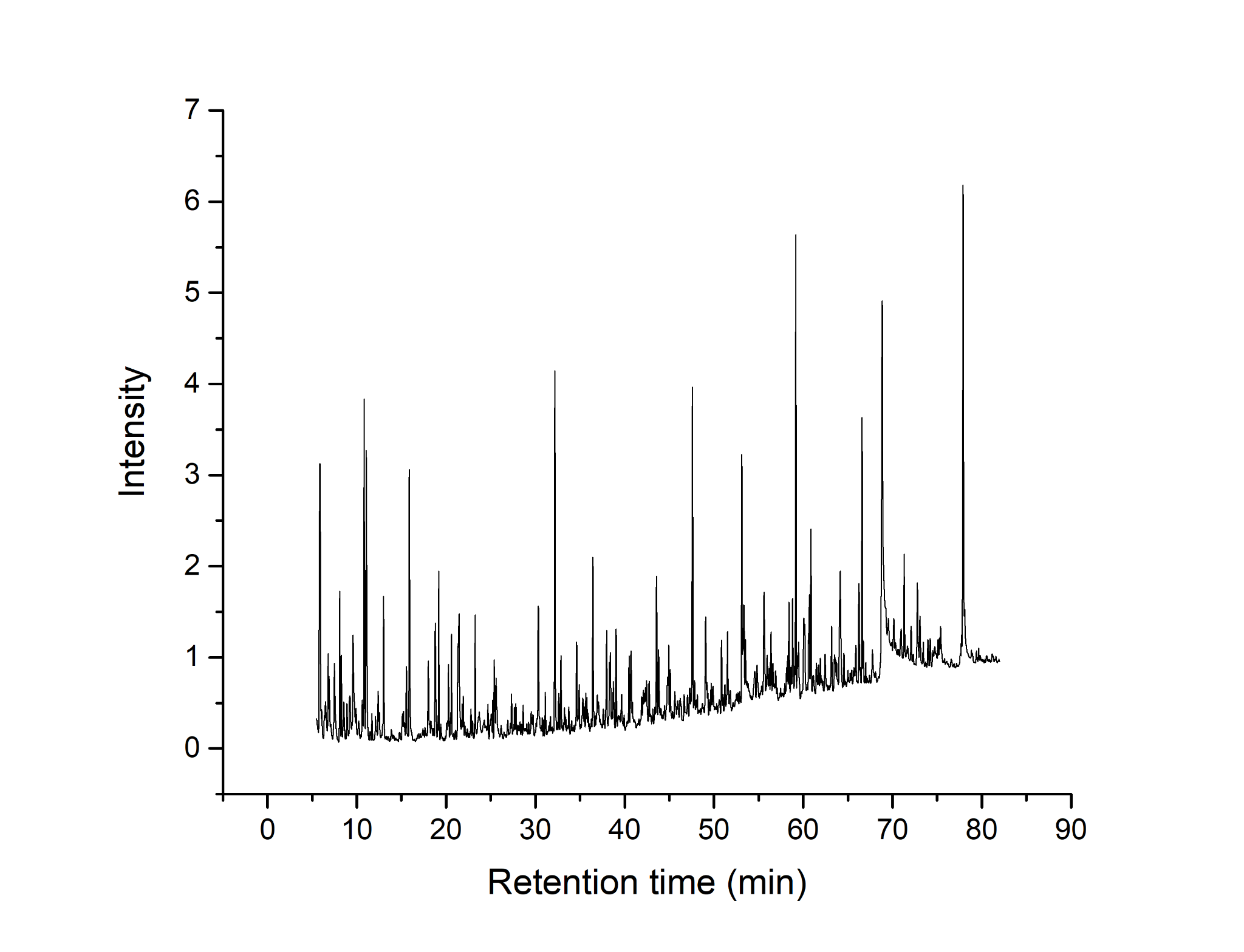
**

**S15 Fig - Pyrograms of volatile compounds analysis by GC/MS from pyrolysis of sample M5 at 550°C (1).**

**
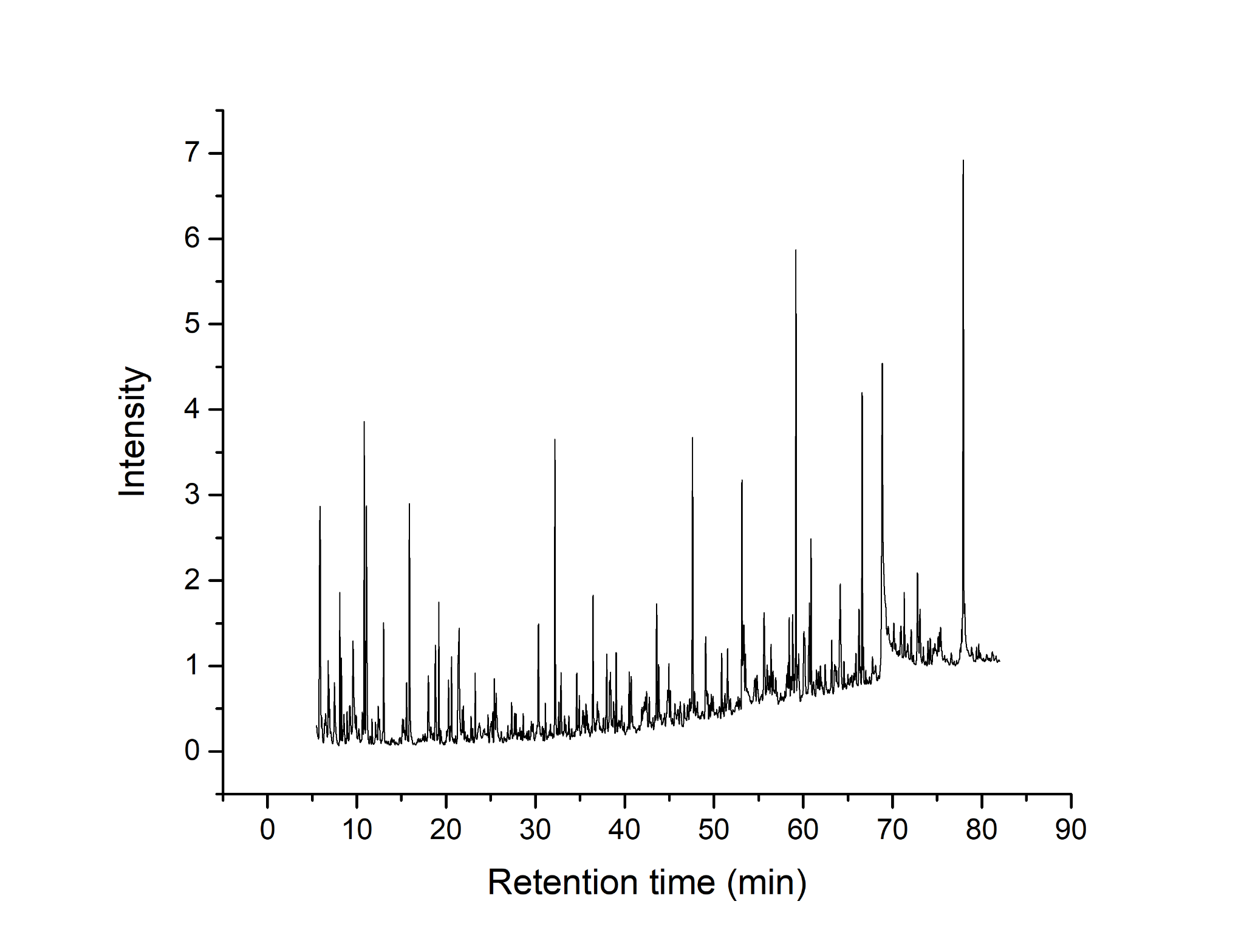
**

**S16 Fig - Pyrograms of volatile compounds analysis by GC/MS from pyrolysis of sample M5 at 550°C (2).**

**
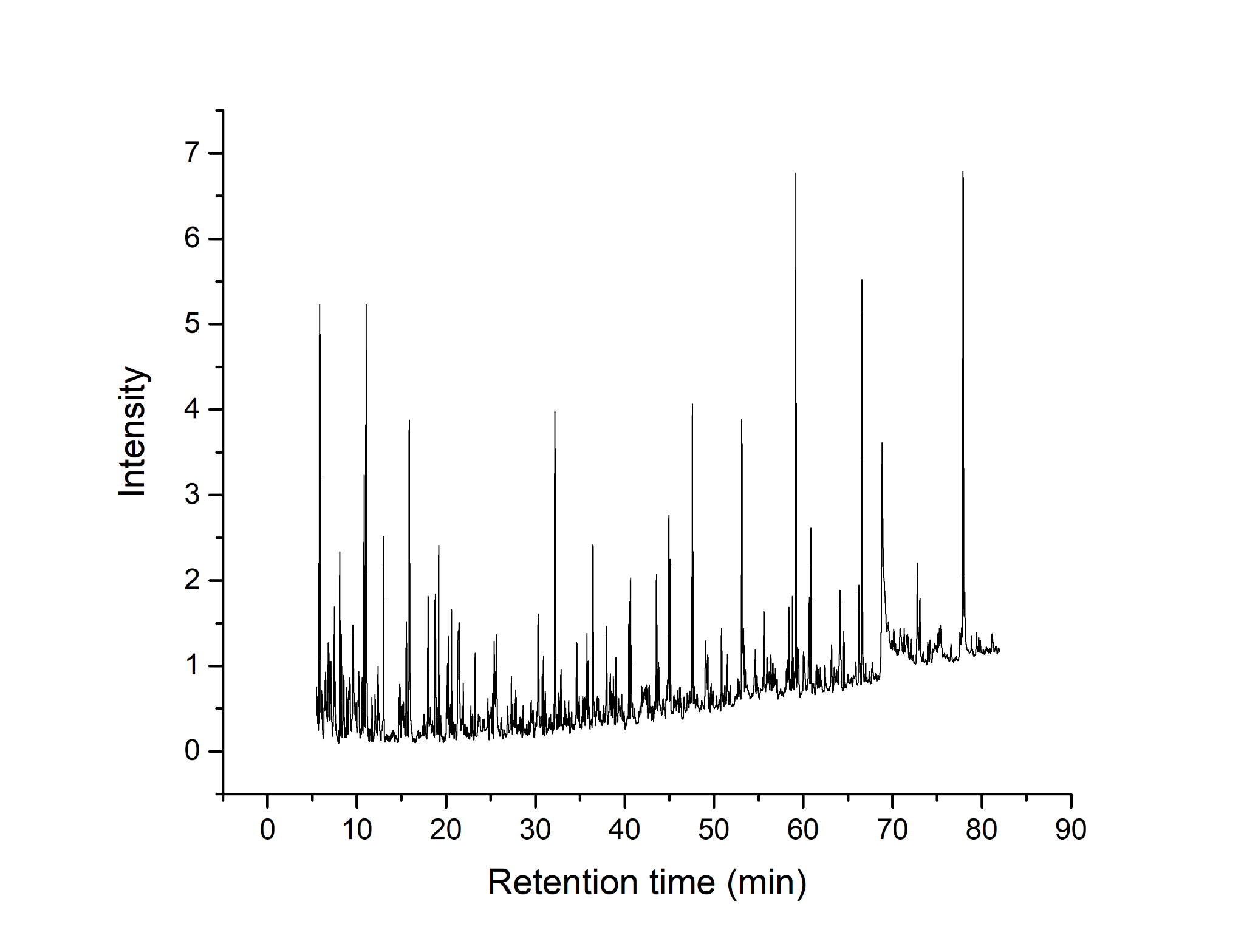
**

**S17 Fig - Pyrograms of volatile compounds analysis by GC/MS from pyrolysis of sample M5 at 650°C (1).**

**
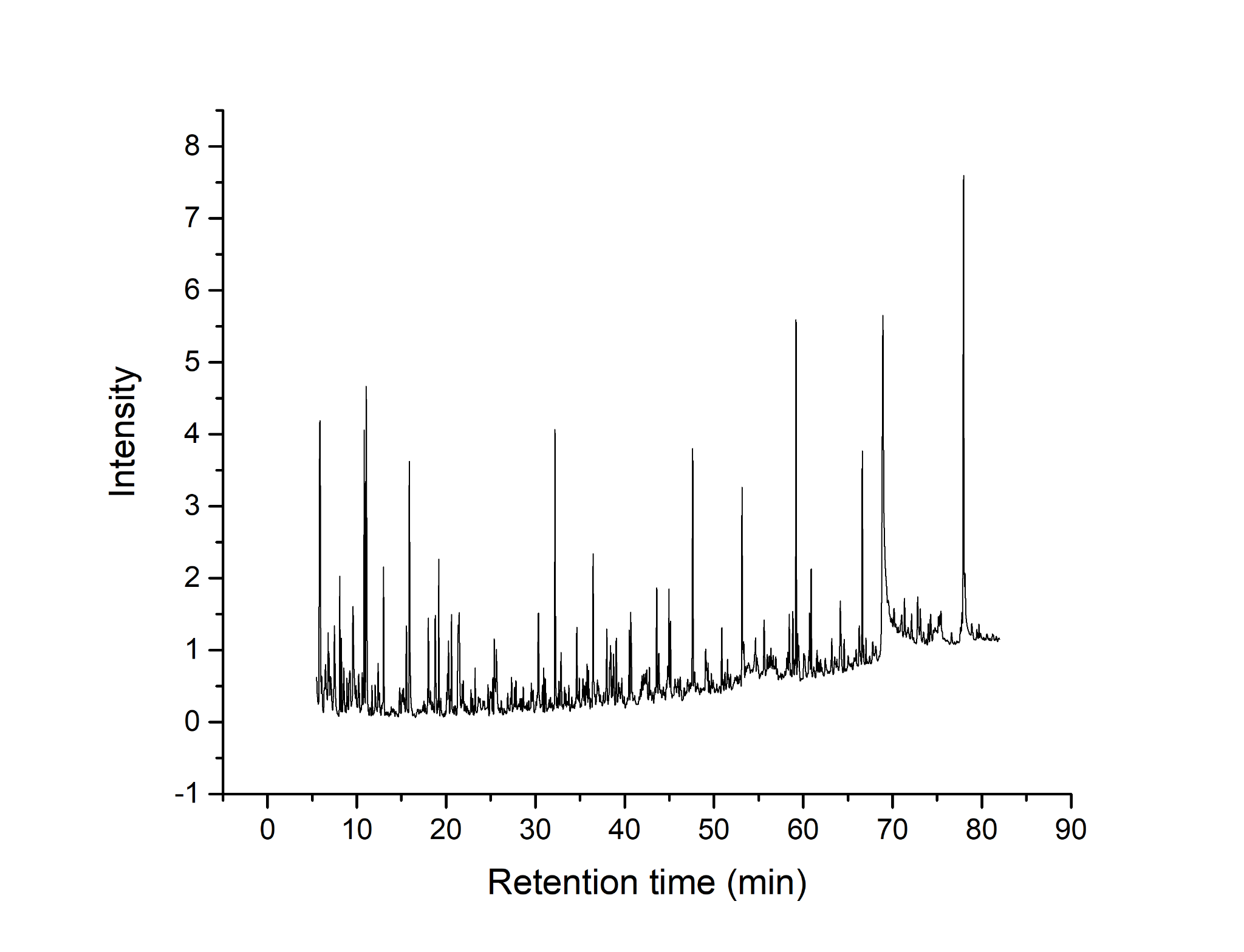
**

**S18 Fig -: Pyrograms of volatile compounds analysis by GC/MS from pyrolysis of sample M5 at 650°C (2).**

**
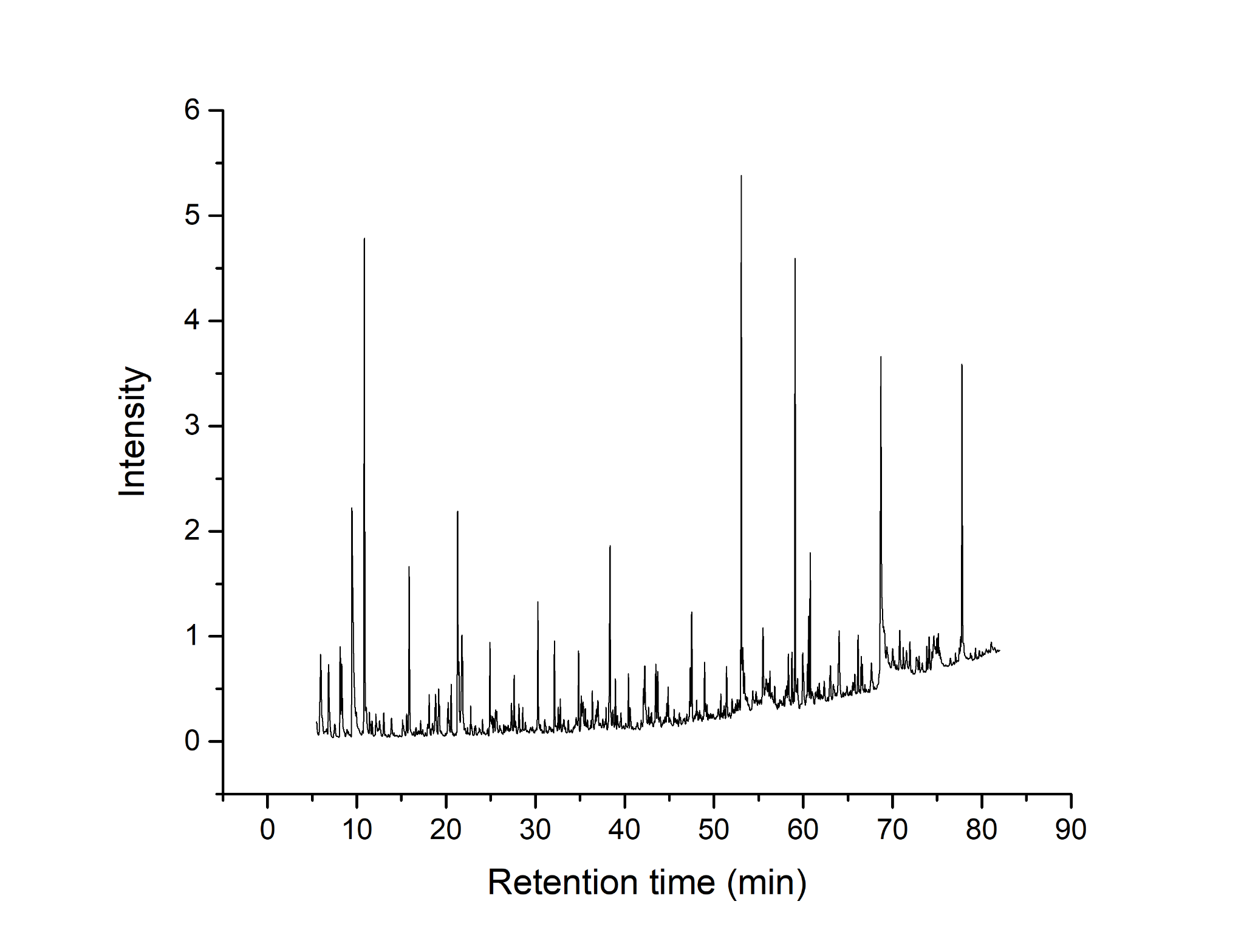
**

**S19 Fig - Pyrograms of volatile compounds analysis by GC/MS from pyrolysis of sample M10 at 450°C (1).**

**
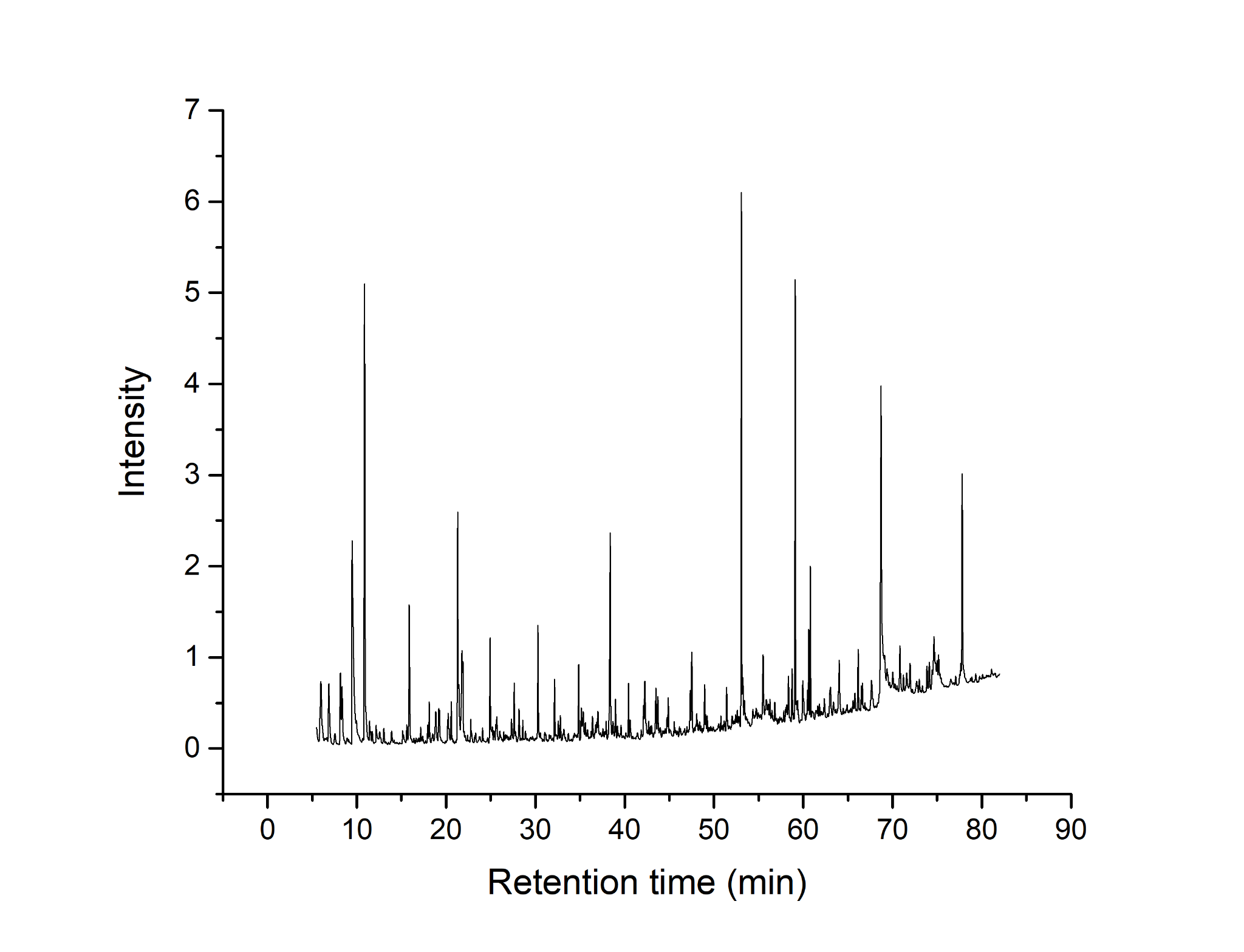
**

**S20 Fig - Pyrograms of volatile compounds analysis by GC/MS from pyrolysis of sample M10 at 450°C (2).**

**
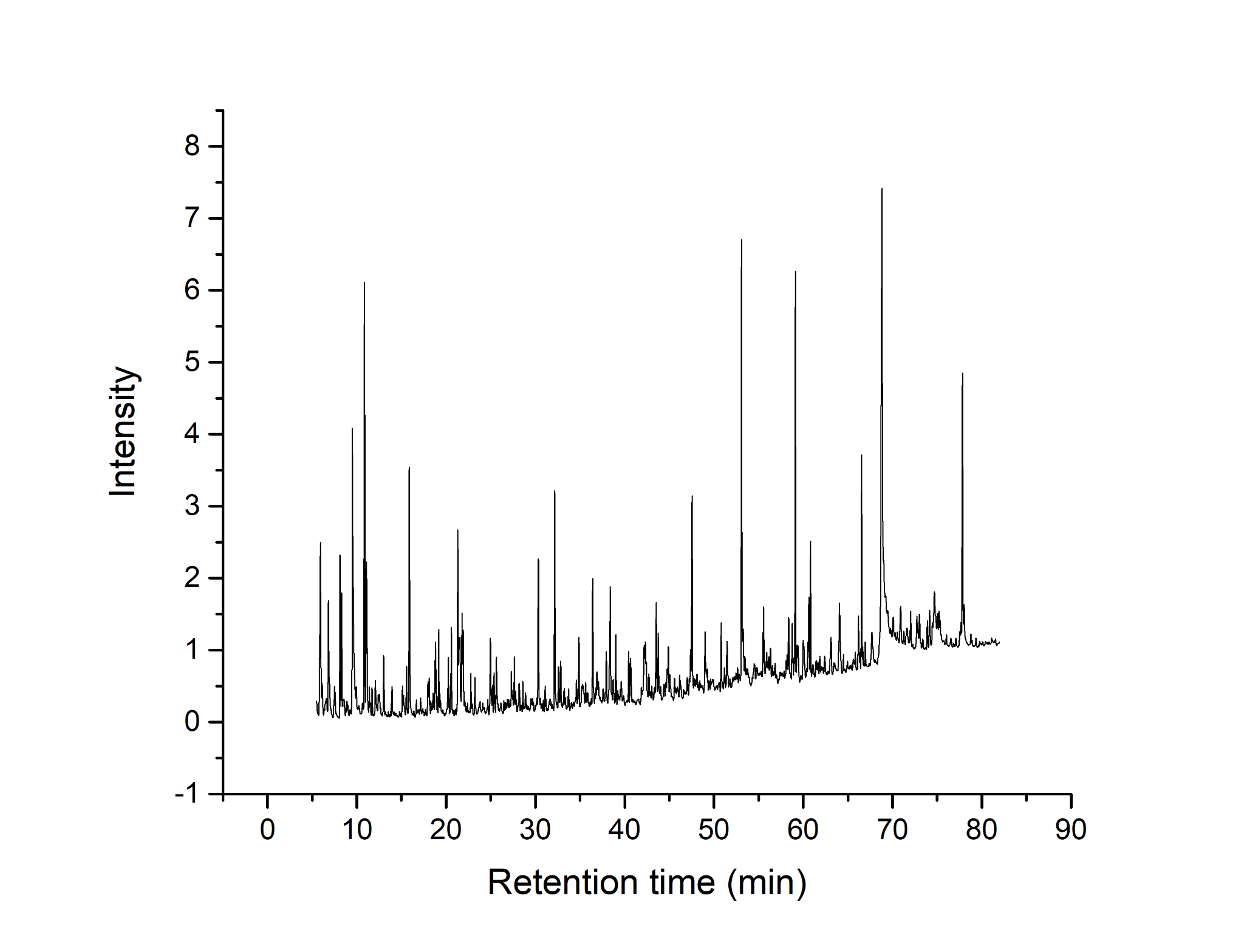
**

**S21 Fig - Pyrograms of volatile compounds analysis by GC/MS from pyrolysis of sample M10 at 550°C (1).**

**
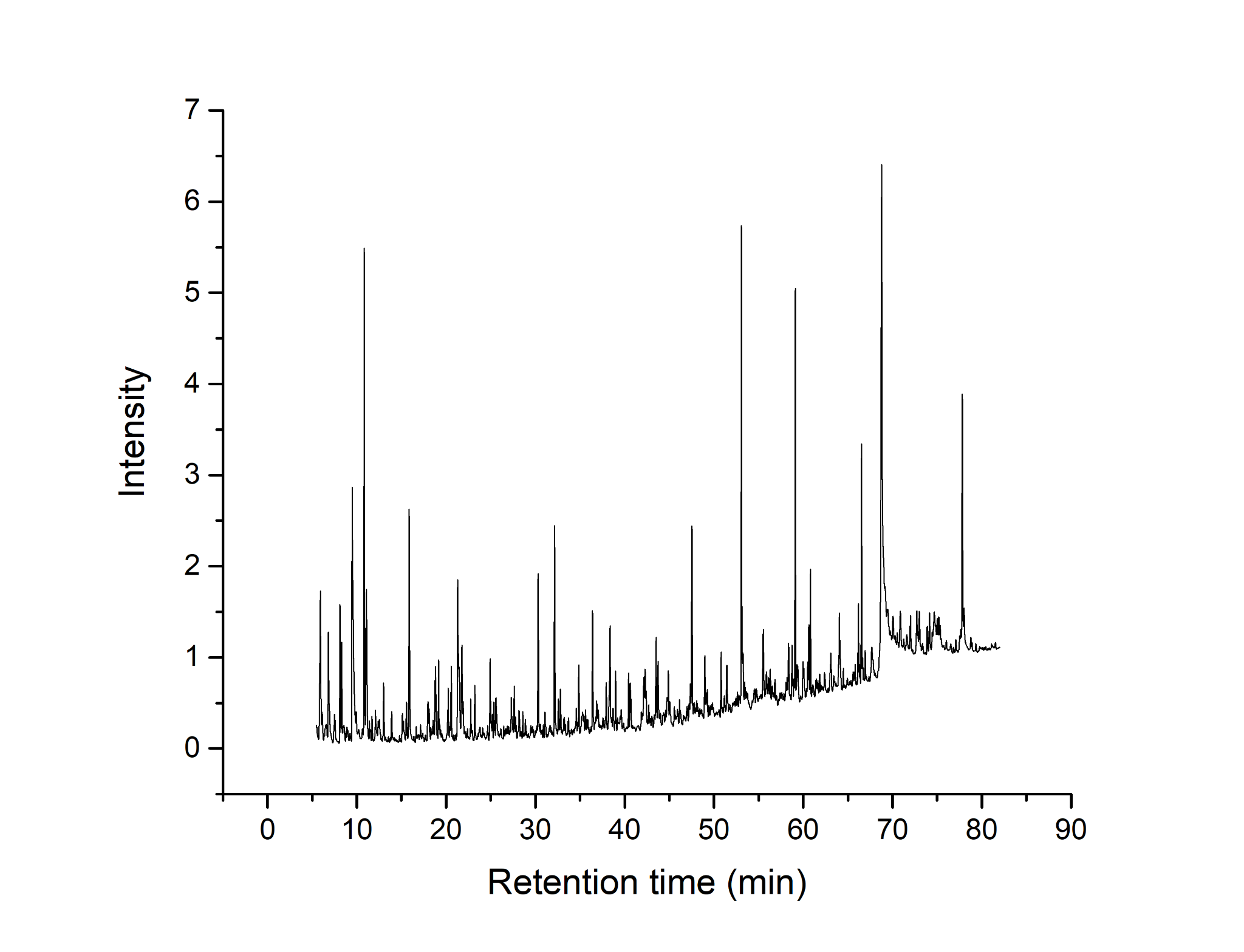
**

**S22 Fig - Pyrograms of volatile compounds analysis by GC/MS from pyrolysis of sample M10 at 550°C (2).**

**
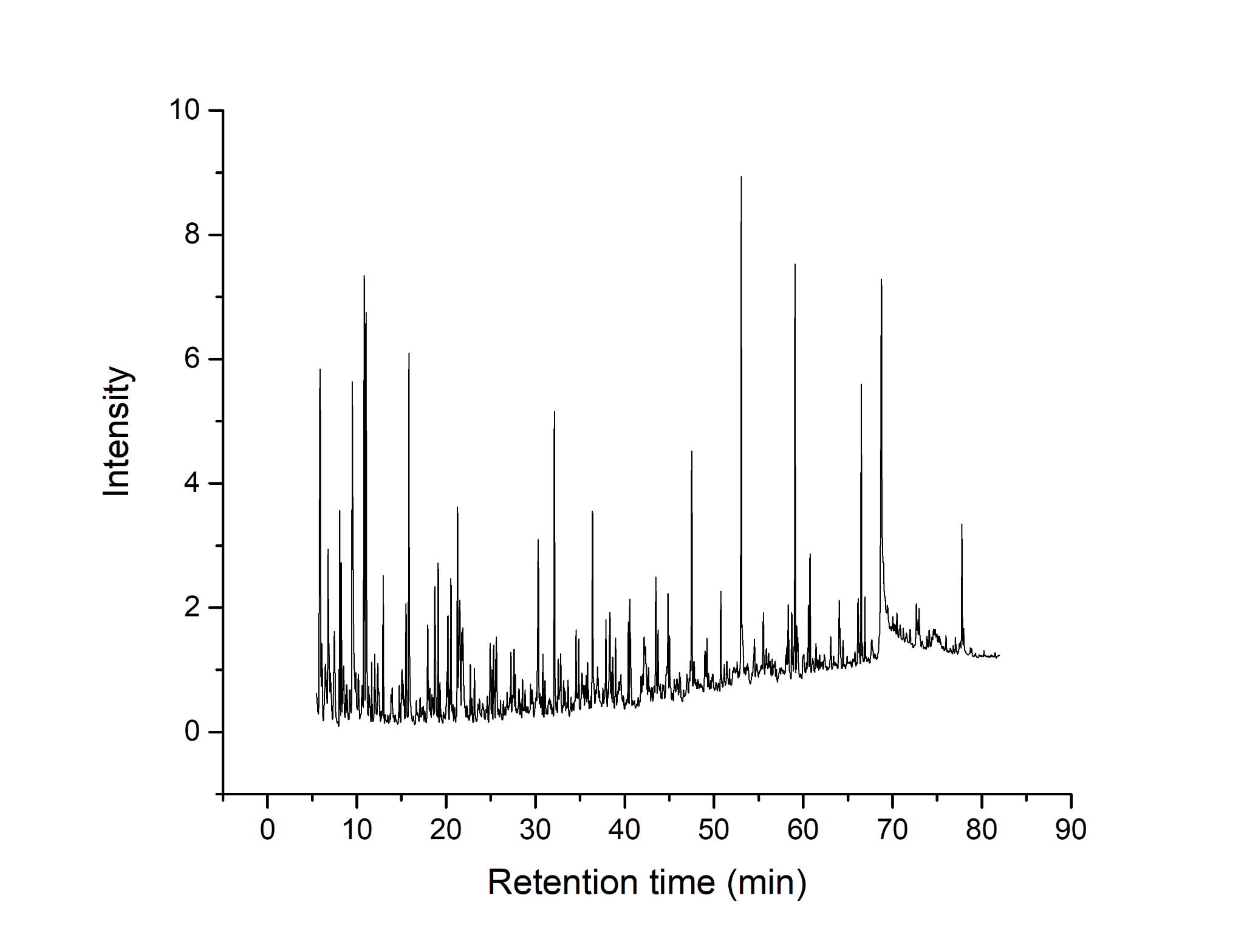
**

**S23 Fig - Pyrograms of volatile compounds analysis by GC/MS from pyrolysis of sample M10 at 650°C (1).**

**
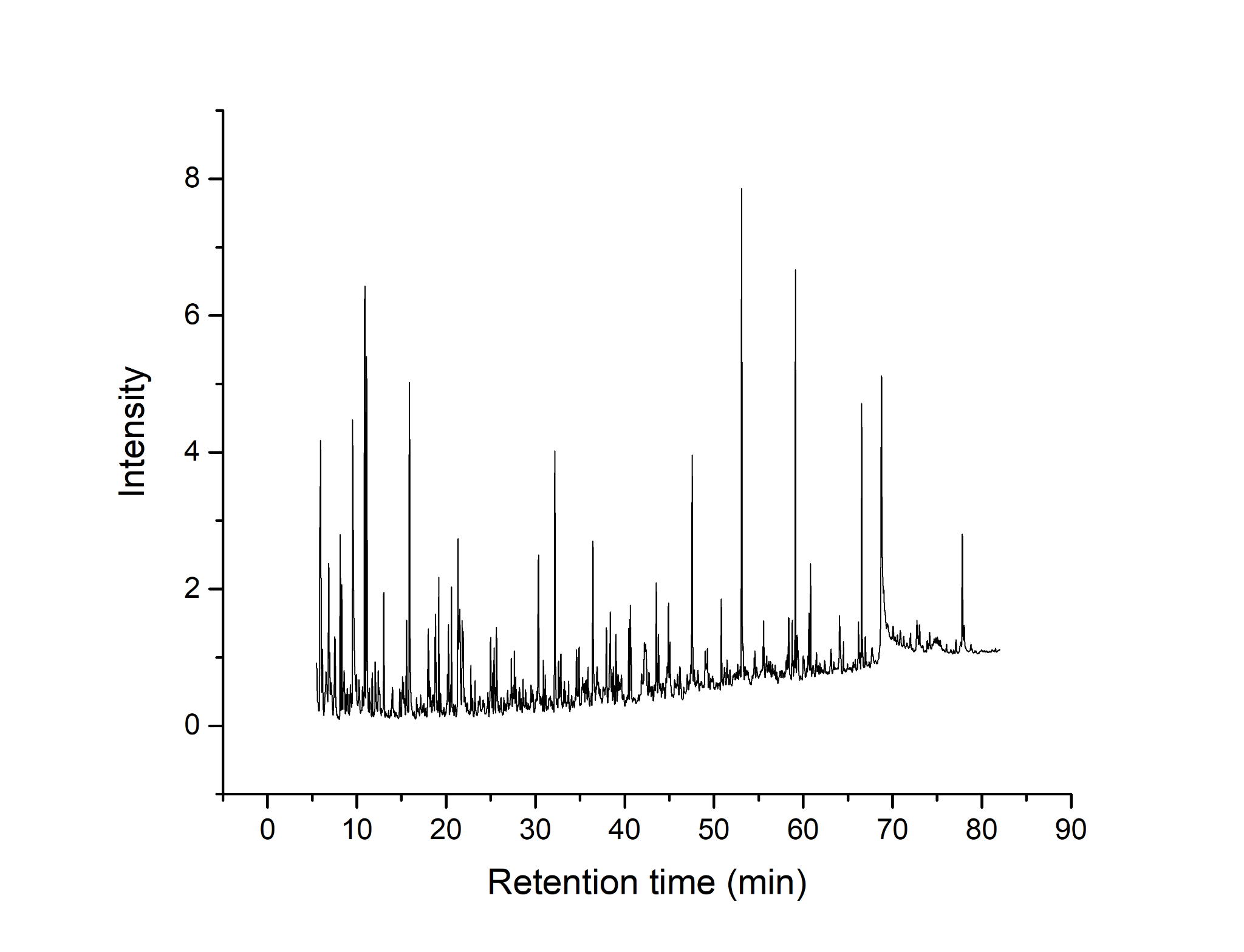
**

**S24 Fig - Pyrograms of volatile compounds analysis by GC/MS from pyrolysis of sample M10 at 650°C (2).**
